# Association of cognitive impairment and peripheral artery disease (PAD): A systematic review

**DOI:** 10.1177/1358863X251336736

**Published:** 2025-05-21

**Authors:** Beth L Cheshire, Sarah J Messeder, Coral J Pepper, Lucy C Beishon, Rob D Sayers, John SM Houghton

**Affiliations:** 1 Department of Cardiovascular Sciences, University of Leicester, Leicester, UK; 2 Leicester Vascular Institute, University Hospitals of Leicester NHS Trust, Leicester, UK; 3 British Heart Foundation, The Glenfield Hospital, Leicester, UK; 4 Library Service, University Hospitals of Leicester NHS Trust, Leicester, UK; 5 National Institute for Health Research Leicester Biomedical Research Centre, The Glenfield Hospital, Leicester, UK

**Keywords:** ankle–brachial index (ABI), atherosclerosis, cognitive impairment, dementia, peripheral artery disease (PAD)

## Abstract

This systematic review aimed to describe the association between peripheral artery disease (PAD) and cognitive impairment or dementia. We included studies reporting on the association between PAD (defined by ankle–brachial index ⩽ 0.9) and cognitive function in adult populations. MEDLINE, Embase, CINAHL, PsycINFO, and CENTRAL were systematically searched from inception to January 2025. Study quality was assessed using the Risk of Bias In Nonrandomized Studies of Exposure effects (ROBINS-E) tool. A narrative synthesis was undertaken structured by cognitive outcome and study design. Thirty-eight studies were included in the review (58,586 participants). The results provide evidence that PAD is associated with cognitive impairment. Seventeen (81%) cross-sectional studies and four (67%) longitudinal studies reported associations of PAD with poorer cognitive performance or increased risk of cognitive impairment. Impaired memory and processing speed were most frequently associated with PAD. PAD was also associated with increased dementia risk in cross-sectional (odds ratios = 1.50–2.41) and longitudinal studies (hazard ratios = 1.03–2.40), although proportionally fewer longitudinal studies reported significant association of PAD with dementia. Results suggest increased prevalence of cognitive impairment, independent of cardiovascular risk factors and cerebrovascular disease. Awareness of the prevalence of cognitive impairment and its potential impact on treatment adherence and engagement in a healthy lifestyle is important for clinicians treating patients with PAD. Screening for cognitive impairment in those with PAD may aid early diagnosis and management of cognitive impairment in this high-risk population. Further research is required to determine whether screening for and optimal management of PAD has cognitive benefits. **(PROSPERO Registration No.: CRD42023399608)**

## Introduction

Cognitive impairment is a spectrum of diseases from mild cognitive impairment (MCI) to dementia and there exist various subtypes that have different clinical presentations and etiologies. MCI is defined as a decline in cognitive functioning beyond that expected for an individual’s age and education level, but that does not interfere with daily living.^1^ It is often described as the intermediate phase between normal cognitive aging and the cognitive deterioration observed in dementia, with risk of dementia progression higher in those with MCI compared to cognitively healthy individuals.^1,2^ The two most common types of dementia are Alzheimer’s disease (AD) and vascular dementia (VaD). AD is characterized by two main neuropathological lesions: extracellular amyloid-β plaques and intraneuronal neurofibrillary tangles of hyperphosphorylated tau proteins. It most commonly presents with memory loss, particularly impaired learning or recall of recent learning, plus dysfunction in one other cognitive domain.^3–7^ Vascular cognitive impairment and dementia are a heterogeneous group of cognitive disorders caused by cerebrovascular disease that result in ischemic or hemorrhagic brain injury and disruption of neurocognitive networks.^6,8^ Impairment in executive function predominates over memory loss and it is characterized by white matter damage (leukokariosis), small vessel disease, and lacunar infarcts, identified on brain imaging.^9,10^ Some individuals may present with mixed Alzheimer’s and vascular type cognitive impairment and recent evidence suggests that there may be an underlying vascular component to the etiology of Alzheimer’s dementia.^10–14^

Cardiovascular disease (CVD) is a major risk factor for the development of cognitive impairment and dementia.^15,16^ They also share a number of risk factors, including age, diabetes, hypertension, and smoking,^17–20^ and similar pathogenic mechanisms such as ischemia and atherosclerosis.^21^ Cardiovascular risk factors (CRFs) increase the risk of cognitive impairment and dementia potentially through cerebral hypoperfusion, embolism, hypoxia, or infarcts, resulting in degenerative and vascular brain lesions.^21,22^ Peripheral artery disease (PAD) is strongly associated with an increased risk of CVD and all-cause and cardiovascular mortality.^23^ Though most individuals with PAD are asymptomatic, the disease may progress, leading to symptoms of intermittent claudication, pain at rest, ulceration, and gangrene.^24^ Diagnosis of PAD is confirmed with hemodynamic tests, the standard being the ankle–brachial index (ABI),^25^ with values of < 0.90 used to diagnose PAD.^26^ First-line management of PAD is optimal medical therapy, exercise, and lifestyle modifications such as smoking cessation which aims to reduce cardiovascular risk.^24,27^

There is some evidence of an increased prevalence of cognitive impairment, independent of CRFs and previous cerebrovascular disease, in people with PAD.^28^ Among vascular surgery patients, the prevalence of cognitive impairment may be as high as 50%.^29^ A narrative review of evidence of the association between PAD and cognition revealed that patients with PAD generally have worse cognitive function than controls, and in population-based studies PAD was associated with greater cognitive decline.^28^ A systematic review of the relationship between ABI and cognitive impairment in the general population provides further evidence of an association between low ABI and cognitive impairment and dementia, with all reviewed studies except one reporting a significant association.^30^ A more recent review on impaired oxygen supply and cognitive impairment in PAD also revealed impaired cognitive function in patients with PAD.^31^

Modifiable risk factors account for 45% of dementia cases worldwide, with 17% of the attributable risk for dementia accounted for by CRFs.^8,32^ Many CRFs are modifiable through early identification, treatment, and healthier lifestyles, with improvements in vascular health potentially reducing the risk of cognitive impairment.^33,34^ Identifying clinical markers that predict cognitive impairment would be invaluable in aiding early detection and treatment in order to delay or prevent further decline. Evidence of an association between PAD and cognitive impairment would suggest that PAD is a risk factor for the development of cognitive impairment and that screening using ABI may be useful in predicting cognitive decline.^35,36^

The aim of this systematic review was to undertake a comprehensive investigation of the association between PAD diagnosed by ABI and cognitive impairment. Secondary aims were to assess which cognitive domains are predominantly impaired in patients with PAD and to investigate associations of PAD with both MCI and dementia.

## Methods

The review was performed according to Preferred Reporting Items for Systematic reviews and Meta-Analyses (PRISMA) guidelines^37^, and the protocol was registered with the International Prospective Register of Systematic Reviews (**PROSPERO Registration No.: CRD42023399608**).

### Search strategies

Electronic databases including MEDLINE, Embase, the Cumulative Index to Nursing and Allied Health Literature (CINAHL), PsycINFO (including PsycArticles), and the Cochrane Central Register of Controlled Trials (CENTRAL) were systematically searched for articles assessing PAD and cognitive function in adult populations. Initial searches were undertaken from inception to January 2023, with searches updated on January 29, 2025. The review was managed in Covidence (Covidence systematic review software, Veritas Health Innovation), including combining and de-duplicating search results from each database, screening, and study selection. The reference lists of included studies and identified relevant review articles were manually searched. Searches for published studies from conference abstracts were conducted and where identified were included in the review. The search strategies were designed in collaboration with an experienced clinical librarian (CP) in MEDLINE and adapted for, and applied to, the other databases. Full search strategies are detailed in Supplemental Appendix.

### Study selection

Titles and abstracts were independently screened by both BC and a second reviewer (JH or SM) according to the inclusion and exclusion criteria (Supplemental Table S1). Full texts were then retrieved and independently screened by two reviewers (BC and JH or SM) with disagreements resolved by discussion. PAD was defined as an ABI ⩽ 0.9. Studies that assessed cognitive function, impairment, or dementia using any validated method (e.g., screening tool, neuropsychological assessment, clinical diagnostic criteria) were included. In cases where duplicate publications from the same cohort and wave were identified, only the most relevant were included. Multiple publications from the same cohorts were included if the data were from different timepoints or the outcome (e.g., cognitive function vs dementia) differed.

### Data extraction and quality assessment

Data were extracted from the selected studies independently by both BC and a second reviewer (JH or SM) including: author, year of publication, country, study design, sample size, summary age and sex statistics, recruitment date, and follow-up duration (where applicable). The proportion of individuals with PAD, cognitive outcome and assessments/criteria, exclusion criteria, and study results were also extracted. Study quality was assessed using the Risk of Bias In Nonrandomized Studies of Exposure effects (ROBINS-E) tool^38^ independently by the same reviewers with disagreements resolved by discussion. Studies were not excluded on the basis of quality or risk of bias. However, quality and bias were considered when synthesizing results. Risk of bias due to confounding was assessed based on factors associated with cognitive impairment. Studies that controlled for all the following factors in their analysis model were considered well-adjusted: age, education (or intelligence), diabetes, hypertension (or blood pressure), smoking, and stroke. Studies that controlled for all but one of these factors were also considered well-adjusted, provided they included age, education, and stroke in the analysis model.

### Narrative synthesis

A narrative synthesis of results was undertaken for all included studies and data were summarized in tables. Owing to methodological and statistical heterogeneity, meta-analysis of aggregate data was not possible. The narrative synthesis was structured in four separate sections by cognitive outcome (cognitive function/impairment or dementia) and study design (longitudinal or cross-sectional). The methods used to assess cognitive impairment (e.g., neuropsychological tests, screening tools, and clinical diagnosis) in the included studies, and results by cognitive outcome, were evaluated. For studies assessing various cognitive functions, neuropsychological tests were categorized according to the primary cognitive domain, and results synthesized by separate cognitive functions. These included language ability, verbal fluency, visuospatial ability, short- and long-term memory, processing speed, sensory/motor function, and aspects of executive function including inhibition, selective or sustained attention, cognitive flexibility, abstract reasoning, and working memory.

## Results

**Figure 1.**
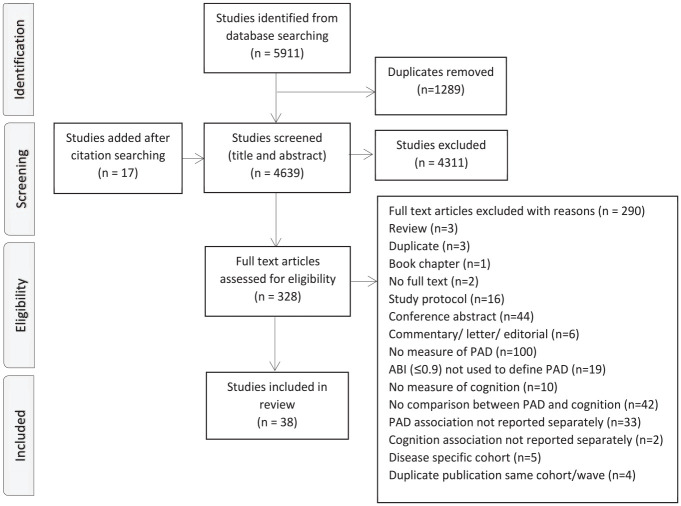
Preferred Reporting Items for Systematic reviews and Meta-Analyses (PRISMA) flow diagram of study selection.

After screening 4639 records, a total of 38 studies were included in the review^35,36,39–74^ (Figure 1). Of these, 20 studies primarily aimed to investigate the association between PAD and cognitive function, impairment, or dementia.^35,36,40,45,48–51,53,55,57–59,64,66,67,69–71,74^ The remaining studies reported assessments of both PAD and cognition or dementia but their association was not the primary objective.

### Study design

Detailed study information including cohort and country, design, sample size, sex and age, proportion with PAD, and outcome measure are presented in Table S2. Briefly, 27 studies were prospective, population-based cohort studies;^35,36,39–43,45–47,50–52,54–58,60,62,63,65,66,68,69,72,73^ however, 15 included cross-sectional analyses only.^35,39,41–43,45,46,50,51,54,57,66,69,72,73^ One study was a prospective cohort of individuals referred for an exercise test.^53^ Of the remaining 10 included studies, nine were cross-sectional,^44,49,59,61,64,67,70,71,74^ of which six were case-controlled^49,59,64,67,71,74^ and one was a randomized controlled trial (RCT).^48^ Three studies assessed both cross-sectional and longitudinal associations between PAD and cognitive function, impairment, or dementia.^40,47,48^

Age restrictions to inclusion criteria of participants varied considerably in included studies. The majority of studies included older adults with ages ranging between 50 and 100 years,^36,39–60,62–65,67–72,74^ four used lower minimum age restrictions of 40–45 years^35,61,66^ and 18 years.^73^ Age at baseline is reported for the majority of prospective studies with follow ups ranging from 2 to 18 years. The total number of participants in the included studies was 72,693; however, after excluding potential duplicates from multiple publications in the same cohorts,^36,41,45–47,50,52,54–56,62,68^ data were included from a minimum of 58,586 participants.

Twenty-nine studies used ABI cut-offs of < 0.9 or ⩽ 0.9 to define PAD.^35,36,39,41–45,48–52,54,56–59,61–63,65–68,70–72,74^ Four studies used slightly lower^40,46,47,64^ ABI cut-off values, and five analyzed ABI as a continuous variable.^53,55,60,69,73^

The assessment method for cognitive function and impairment varied among included studies and is detailed in Tables 1–4. Fourteen studies examined cognitive function using neuropsychological test batteries, with continuous scores used to indicate level of performance or decline.^36,39,40,48,49,51,52,55,57,59,61,64–66^ Eleven studies assessed global cognitive function using screening assessments or diagnostic criteria: two analyzed scores as continuous variables^44,71^ and nine used cut-off scores to indicate impairment.^35,41,43,46,47,69,70,73,74^ The most frequently used screening test was the Mini-Mental State Examination (MMSE): five studies used standardized thresholds of < 24 to indicate cognitive impairment.^35,41,43,70,73^

**Table 1.** Cross-sectional association between peripheral artery disease (PAD) and cognitive function and impairment.

Study	Cognitive domains	Threshold	Result	Adjustment^a^	Exclusion
André-Petersson (2001)^39^	LA, VSA, PS, STM	^b^	No difference in cognitive scores between low (< 0.9) and normal ABI (*p* > 0.05)	Unadjusted Age^c^, sex^c^	—
Bareiro (2024)^40^	Global (MMSE), VF, STM, LTM, VSA, EF^○ △^	^b^	Declining ABI associated with lower MMSE scores in < 0.8 category (*p* = 0.048) and decreasing STM and VSA in each ABI category (1.4 to > 1.1; < 1.1 to 0.8; < 0.8) (*p* = 0.03 − 0.005)	Age, sex, DM, HTN, Edu, Smk	Dem, MI, AP, IC, TIA, stroke, ABI > 1.4
Breteler (1994)^41^	Global (MMSE)	< 24 < 26	Higher proportion of participants with MMSE scores < 24 and < 26 with PAD (ABI < 0.9) compared to without (10.4% vs 5.0% and 17.5% vs 11.1%, *p* < 0.0001)	Age, sex, Edu, Smk^d^	—
Buscemi (2017)^43^	Global (MMSE) VSA (CDT)	⩽ 24 ⩽ 4	Lower ABI in those with MCI compared to without (M [SD]: 1.05 [0.08] vs 1.07 [0.07], *p* < 0.05). ABI positively correlated with VSA (*p* = 0.03), but not MMSE scores.	Unadjusted Age^c^, Edu^c^	LDL, statins
Chen (2018)^44^	Global (AD8)	^b^	No association between ABI and AD8 scores (*p* = 0.74)	Unadjusted	—
Chen (2024)^73^	Global (MMSE)	< 24	No difference in ABI between those with cognitive impairment and those without (median: 1.08 vs 1.11, *p* = 0.172)	Unadjusted	—
Di Carlo (2000)^46^	Global (MMSE, CAMDEX)	< 24 CIND ARCD	No difference in PAD frequency between those with CIND or ARCD and cognitively normal (*p* > 0.05)	Unadjusted	Dem
Di Carlo (2007)^47^	Global (MMSE), STM, LTM, EF^△^	CIND MCI	Higher frequency of PAD in those with single nonmemory CIND compared to cognitively normal (*p* = 0.034)	Unadjusted Age^c^, Edu^c^	Dem
Espeland (2015)^48^	Global (3MS), PS, STM, LTM, EF^○ △ ◊^	^b^	Low ABI (< 0.9) associated with poorer cognitive performance on all tasks (*p* < 0.0001 to 0.05) except visual working memory (*p* > 0.05) compared to ABI 0.9–1.30 and > 1.30	Age, sex, Edu, DM, HTN, Smk	Dem
Gardner (2021)^49^	STM, LTM, PS, EF^○ ◊^	^b^	PAD associated with poorer performance on all cognitive tasks compared to controls (unadjusted *p* < 0.001). Final model, PAD associated with poorer STM (*p* = 0.022; *p* < 0.001), LTM (*p* < 0.001), and working memory (*p* = 0.003).	Age, sex, Edu, Smk, CeVD, HTN, DM	Dem, ND, AP, cancer, CKD, HF
Guo (2023)^74^	Global (MMSE, MOCA), STM, LTM, LA, VSA, EF^◊^	^b^ Jak/Bondi criteria	Low ABI (⩽ 0.9) associated with cognitive decline (OR = 3.72 [CI: 2.39–7.53], *p* = 0.003) adjusted for smoking history. Fully adjusted, ABI independently associated with cognitive decline (OR = 2.96 [CI: 1.87–6.46], *p* = 0.037). ABI correlated with memory (*r* = 0.39; *p* = 0.006) and VSA (*r* = 0.51; *p* < 0.001).	Age, sex, Smk, HTN, DM	MCI, Dem, CeVD, Alc/SubA, Dep, ABI > 1.4
Gutierrez (2015)^51^	PS	^b^	PAD associated with poorer cognitive performance compared to those without in the unadjusted model only	Age, sex, Edu, HTN, Smk, DM, stroke	INSTD
Laukka (2014)^57^	Global, STM, LTM, VF, PS, EF^◻ ○ △^	^b^	Lower ABI associated with worse global ability (*p* = 0.04), working memory (*p* < 0.01), PS (*p* = 0.01; *p* = 0.04), and better STM and LTM (*p* = 0.01). Fully adjusted, higher ABI associated with better global ability (*p* = 0.02) and PS (*p* < 0.01).	Age, sex, IQ, CeVD, Smk^d^, HTN^d^, DM^d^	Dem
Mangiafico (2006)^59^	STM, LTM, VSA, PS, EF^○ ◊^	^b^	Individuals with APAD performed worse than controls on all cognitive tasks (*p* < 0.0001) except for STM	Unadjusted Age^c^, sex^c^, Edu^c^	Stroke, TIA, ND, PsyD, Alc/SubA, cancer, DM,^e^ HTN^e^
Muller (2007)^61^	STM, LTM, VF, PS, EF^○ ◊^	^b^	Participants with ABI < 0.9 had a 0.60 lower score on a memory task only. No association between ABI (< 0.9) and composite memory, PS, or EF scores.	Age, Edu, sex^c^	INSTD
Phillips (1997)^64^	VF, STM, LTM, PS VSA, LA, MF, EF^◻ ○ ◊^	^b^	Patients with PAD performed worse than controls on measures of abstract reasoning and cognitive flexibility, LTM, PS, and VSA (*p* < 0.0001 to *p* < 0.001)	Unadjusted Age^c^, Edu^c^	Stroke, PsyD, ND, Alc/SubA
Tarraf (2018)^66^	Global (SIS), STM, LTM, VF, PS, global composite	SIS ⩽ 4 ^b^	ABI not associated with risk of low mental status (*p* > 0.05). ABI associated with all cognitive measures in unadjusted and fully adjusted linear and quadratic models (*p* < 0.001 to *p* < 0.05). PAD (ABI ⩽ 0.9) associated with lower scores on all tasks compared to ABI (1–1.39), in unadjusted (*p* < 0.001) and fully adjusted models (*p* < 0.05).	Age, sex, Edu, DM, HTN, Smk	Stroke, TIA, CHD
Wang (2016)^35^	Global (MMSE)	< 24	Low ABI (< 0.9) associated with increased risk of cognitive impairment (OR = 2.91 [CI: 1.77–4.80]) and continuous ABI (OR = 1.22 [CI: 1.07–1.39]). Fully adjusted, associations remained for categorical (OR = 1.98 [CI: 1.15–3.42]) and continuous ABI (OR = 1.16 [CI: 1.01–1.32]).	Age, sex, Edu, Smk, HTN, DM	Stroke, TIA, CHD, ND, ABI ⩾ 1.40
Weimar (2015)^69^	STM, LTM, PS, VF, VSA	IWGMCI aMCI naMCI	Decreasing ABI associated with higher prevalence of MCI (PR = 1.11 [CI: 1.07–1.15], *p* < 0.001), aMCI (PR = 1.11 [CI: 1.03–1.20], *p* < 0.01), and naMCI (PR = 1.20 [CI: 1.11–1.29], *p* < 0.001) in unadjusted models. Fully adjusted models, associations remained for MCI (PR = 1.06 [CI: 1.01–1.11], *p* = 0.012) and naMCI (PR = 1.12 [CI: 1.03–1.21], *p* = 0.006).	Age, sex, Edu, DM, stroke, Smk, SBP, DBP, HTN	Dem, ABI > 1.5
Woo (2006)^70^	Global (MMSE)	< 24	Cognitive impairment positively associated with low ABI (< 0.9) (OR= 1.75 [CI: 1.33–2.30]) age-sex adjusted, and remained significant in the fully adjusted model (OR = 1.58 [CI: 1.19–2.09])	Age, sex, DM, HTN, Smk	—
Zimmermann (2011)^71^	Global (MMSE)	^b^	MMSE scores lower in patients with PAD (M [SD]: 28.19 [1.72]) compared to those without (28.67 [1.56]), *p* < 0.001	Unadjusted	Dem, LER, LEA, ABI > 1.30, INSTD

a Only confounders considered in the quality assessment are listed.

b Cognitive scores analyzed as continuous variables.

c Matched between groups/scores corrected for/or same across cohort.

d Reported further adjustment for factors did not affect the pattern of results.

e Uncontrolled.

*Executive function (EF)*: △, inhibition/selective attention/sustained attention; ◊, cognitive flexibility; ◻, verbal/nonverbal abstract reasoning; ○, working memory.

ABI, ankle–brachial index; AD8, Eight-item informant interview to differentiate aging and dementia; Alc/SubA, alcohol/substance abuse; aMCI, amnestic MCI; AP, angina pectoris; APAD, asymptomatic peripheral artery disease; ARCD, age-related cognitive decline; CAMDEX, Cambridge Mental Disorders of the Elderly Examination; CDT, clock drawing test; CeVD, cerebrovascular disease; CHD, coronary heart disease; CIND, cognitive impairment no dementia; CKD, chronic kidney disease; DBP, diastolic blood pressure; Dem, dementia (clinical diagnosis/MMSE scores < thresholds); Dep, depression; DM, diabetes mellitus; Edu, education; HF, heart failure; HTN, hypertension; IC, intermittent claudication; INSTD, institutionalized; IWGMCI, International Working Group on Mild Cognitive Impairment; IQ, intelligence quotient; LA, language ability; LDL, low-density lipoprotein; LEA, lower-extremity amputation; LER, lower-extremity revascularization; LTM, long-term memory; M [SD], mean [SD]; MCI, mild cognitive impairment; MF, motor function; MI, myocardial infarction; MMSE, Mini-Mental State Examination; MOCA, Montreal Cognitive Assessment; 3MS, Modified Mini-Mental State; naMCI, nonamnestic MCI; ND, neurological disorder; OR, odds ratio; PR, prevalence ratio; PS, processing speed; PsyD, psychiatric disorder; SBP, systolic blood pressure; SIS, Six-Item Screener; Smk, smoking; STM, short-term memory; TIA, transient ischemic attack; VF, verbal fluency; VSA, visuospatial ability.

**Table 2.** Cross-sectional association between peripheral artery disease (PAD) and dementia.

Study	Cognitive assessment	Diagnosis	Criteria	Result	Adjustment^a^	Exclusion
Bruunsgaard (1999)^42^	MMSE	Mild, moderate–severe dementia	CDR WHO ICD-10	No association between ABI (< 0.9 vs ⩾ 0.9) and cognitive status (normal, mild, moderate–severe dementia) (*p* = 0.70)	Unadjusted Age^b^	Ankle BP > 220 mmHg
Desormais (2018)^45^	CSI-D NPB CDR	MCI Dementia	DSM-IV Petersen’s criteria MCI	Low ABI (⩽ 0.9) associated with increased risk of cognitive impairment (MCI or dementia) compared to normal ABI in unadjusted (OR = 1.84 [CI: 1.29–2.63]) and adjusted analysis (OR = 1.52 [CI: 1.03–2.25])	Age, sex, Edu, Smk	Severe comorbidities
Guerchet (2013)^50^	CSI-D NPB CDR	Dementia AD VaD	DSM-IV NINCDS-ADRDA	PAD (ABI ⩽ 0.9) associated with prevalent dementia in unadjusted (OR = 2.43 [CI: 1.44–4.13]) and fully adjusted models (OR = 2.31 [CI: 1.28–4.17])	Age, sex, Edu, HTN, DM, Smk, stroke^c^	Severe comorbidities ABI ⩾ 1.40
Hofman (1997)^54^	MMSE ⩽ 25 GMS-A CAMDEX	VaD AD Other	DSM-III-R NINCDS-ADRDA	PAD associated with VaD (OR = 2.5 [CI: 1.3–4.8]) and all dementia (OR = 1.5 [CI: 1.1–2.0]), but not AD (OR = 1.3 [CI: 0.9–1.8]) or other dementia (OR =1.0 [CI: 0.4–2.4])	Age, sex	—
Tasci (2018)^67^	MMSE CDR	Dementia (all) VaD, AD AD-VaD	DSM-IV NINCDS-ADRDA NINDS-AIREN	Dementia associated with PAD in unadjusted (OR = 2.78 [CI: 1.69–4.60]) and fully adjusted models (OR = 2.41 [CI: 1.34–4.32])	Age, sex, Edu, DM	INSTD, MCI, LEA, LER, ABI > 1.40 Other Dem^d^
Zuliani (2010)^72^	MMSE ⩽ 26 NPB	Dementia	DSM-IV	Individuals with dementia had lower ABI (M [SD]: 0.96 [0.16]) compared to individuals with normal cognition (1.03 [0.19]), *p* = 0.005	Unadjusted	—

a Only confounders considered in the quality assessment are listed.

b Matched between groups/scores corrected for/or same across cohort.

c Reported further adjustment for factors did not affect the pattern of results.

d Dementia with Lewy bodies; Parkinson’s disease dementia; frontotemporal dementia; neuropsychiatric behaviors.

ABI, ankle–brachial index; AD, Alzheimer’s disease; BP, blood pressure; CAMDEX, Cambridge Mental Disorders of the Elderly Examination; CDR, Clinical Dementia Rating scale; CSI-D, Community Screening Interview for Dementia; DM, diabetes mellitus; DSM-III/IV, *Diagnostic and Statistical Manual of Mental Disorders* (3rd/4th Editions); Edu, education; GMS-A, Geriatric Mental State Examination; HTN, hypertension; INSTD, institutionalized; LEA, lower-extremity amputation; LER, lower-extremity revascularization; M [SD], mean [SD]; MCI, mild cognitive impairment; MMSE, Mini-Mental State Examination; NINCDS-ADRDA, National Institute of Neurological and Communication Disorders and Stroke–Alzheimer’s Disease and Related Disorders Association; NINDS-AIREN, National Institute of Neurological Disorders and Stroke–Association Internationale pour la Recherché et l’Enseignement en Neurosciences; NPB, neuropsychological battery; OR, odds ratio; Smk, smoking; VaD, vascular dementia; WHO ICD-10, World Health Organization *International Classification of Diseases* (10th revision).

**Table 3.** Longitudinal association between peripheral artery disease (PAD) and cognitive function and impairment.

Study	Cognitive domains	Threshold	Result	Adjustment^a^	Exclusion
Bareiro (2024)^40^	Global (MMSE), STM, LTM, VSA, VF, MF, EF^○ △^	^b^ (change scores)	Decreasing baseline ABI associated with greater declines over 5 years in STM, LTM, and executive dysfunction (*p* < 0.05) in both the < 1.1 and > 1.1–1.4 ABI categories	Age, sex, DM, HTN, Edu, Smk	Dem^c^, MI^c^, AP^c^, IC^c^, stroke^c^, TIA^c^, ABI > 1.4^c^
Espeland (2015)^48^	Global (3MS), PS, STM, LTM, EF^○ △ ◊^, composite global	^b^ (change scores)	Baseline ABI not associated with 2-year change in cognitive function on any task (*p* > 0.05); neither was change in ABI	Age, sex, Edu, DM, HTN, Smk	Dem^c^
Haan (1999)^52^	Global (3MS), PS	^b^	Low baseline ABI (continuous and dichotomized) associated with greater decline in global function and PS over 7 years (*p* < 0.0001)	Age, sex, Edu, DM, SBP, DBP, stroke^c,d^	—
Johnson (2010)^55^	Global (composite)	^b^	Baseline ABI associated with cognitive function at year 10 (*p* = 0.001). Fully adjusted model, association remained (*p* = 0.03). Baseline ABI or change in ABI (years 5–12) not associated with change in cognitive function between years 10–15 (*p* > 0.05).	Age, sex, Smk, NART	—
Price (2006)^36^	STM, LTM, VF, PS, EF^◻^	^b^ ↓vs↑ tertile	Baseline ABI associated with lower cognitive scores at 10 years, except memory. Fully adjusted, ABI associated with PS (continuous [*p* = 0.001]; dichotomized [*p* ⩽ 0.05]). Low ABI (⩽ 0.95) associated with impaired abstract reasoning, VF, and PS (OR = 1.8–2.2).	Age, sex, NART	Dem, terminal illness
Reijmer (2011)^65^	Composite memory, PS, EF	^b^	Baseline or incident PAD not associated with composite cognitive function scores at 7 years (*p* > 0.05)	Age, sex	—

a Only confounders considered in the quality assessment are listed.

b Cognitive scores analyzed as continuous variables.

c Prevalent at baseline.

d Incident follow up.

*Executive function (EF)*: △, inhibition/selective attention/sustained attention; ◊, cognitive flexibility; ◻, verbal/nonverbal abstract reasoning; ○, working memory.

ABI, ankle–brachial pressure index; AP, angina pectoris; Dem, dementia (clinical diagnosis/MMSE score < thresholds); DBP, diastolic blood pressure; DM, diabetes mellitus; Edu, education; HTN, hypertension; IC, intermittent claudication; LTM, long-term memory; MF, motor function; MI, myocardial infarction; MMSE, Mini-Mental State Examination; 3MS, Modified Mini-Mental State; NART, National Adult Reading Test; OR, odds ratio; PS, processing speed; SBP, systolic blood pressure; Smk, smoking; STM, short-term memory; TIA, transient ischemic attack; VF, verbal fluency; VSA, visuospatial ability.

**Table 4.** Longitudinal association between peripheral artery disease (PAD) and dementia.

Study	Cognitive assessment	Diagnosis	Criteria	Result	Adjustment^a^	Exclusion
Di Carlo (2007)^47^	CAMDEX	Dementia AD VaD	DSM-III-R NINCDS-ADRDA WHO ICD-10 codes	No difference between prevalence of baseline PAD in those who developed dementia (4.2%) compared to those that did not (7.7%) over 3 years	Unadjusted	Dem^b^
Espeland (2015)^48^	N/A	MCI Dementia	NIA-AA	Lower baseline ABI associated with increased risk of poorer cognitive outcome at 2 years (progressing from normal cognition to MCI, or from normal cognition or MCI to probable dementia) (OR = 2.60 [CI: 1.06–6.37]). Individuals with ABI < 0.9 were at greater risk of progressing to MCI (OR = 1.72 [CI: 1.05–2.82]) but not probable dementia (OR = 0.94 [CI: 0.42–2.10]).	Age, sex, Edu, DM, HTN, Smk	Dem^b^
Hietanen (2013)^53^	N/A	Dementia	WHO ICD-9/10 codes	Baseline ABI not different between individuals who developed dementia (M [SD]: 1.20 [0.3]) and those who did not (1.20 [0.3]), over 18 years	Unadjusted	Dem^b^, CVD^b^, stroke^b^
Kuller (2016)^56^	MMSE, IQCODE / DQ, NPB	Dementia	IQCODE DQ	Among White women, low ABI (< 0.9) was related to increased incidence of dementia compared to ABI ⩾ 0.9 (125 [CI: 58–274] per 1000 PY vs 74 [CI: 46–119] per 1000 PY, *p* = 0.020), over 14 years	Age, sex	Dem^b^
Laurin (2007)^58^	CASI	Dementia AD VaD	DSM-III-R NINCDS-ADRDA CADDTC	PAD (ABI < 0.9) associated with increased risk of total dementia (HR = 1.79 [CI: 1.27–2.52]), VaD (HR = 2.79 [CI: 1.38–5.62]), and AD (HR = 1.60 [CI: 1.01–2.53]) at 5 years. Additional adjustment, PAD was associated with increased risk of total dementia (HR = 1.66 [CI: 1.16–2.37]) and VaD (HR = 2.25 [CI: 1.07–4.73]). Fully adjusted, the increased risk remained for total dementia only (HR = 1.62 [CI: 1.13–2.32]).	Age, sex^c^, Edu, HTN, DM, Smk, stroke	Dem^b^
Moon (2015)^60^	CERAD-K, IWG-MCI, NPB	MCI Dementia	IWG-MCI DSM-IV	Baseline ABI lower in individuals who progressed to MCI or dementia over 5 years compared to individuals whose cognitive function remained the same, differences not significant (*p* > 0.05)	Unadjusted	Dem^b^
Newman (2005)^62^	MMSE / 3MSE, NPB, IQCODE, DQ	Dementia AD VaD	NINCDS-ADRDA CADDTC	Incidence of total dementia over 5 years higher in participants with baseline PAD compared to those without (HR = 2.4 [CI: 1.4–4.0]), as was incidence of AD (HR = 2.4 [CI: 1.4–4.2]), and ‘pure AD’ (excluding VaD) (HR = 2.2 [CI: 1.1–4.5])	Age, Edu, HTN^d^, DM^d^, Smk^d^	Dem^b^, MCI^b,e^, stroke^b,e^
Pase (2016)^63^	MMSE, NPB, DSI	Dementia AD	DSM-IV NINCDS-ADRDA	ABI not associated with risk of incident dementia (HR = 1.09 [CI: 081–1.46]) or AD (HR = 1.09 [CI: 0.79–1.52]) at 10 years in any model	Age, sex, Edu, SBP, FSRP	Dem^b^
van Oijen (2007)^68^	MMSE, GMS-A, CAMDEX	Dementia VaD AD	DSM-III-R NINCDS-ADRDA NINDS-AIREN	Baseline PAD not associated with increased risk of dementia (HR = 1.03 [CI: 0.85–1.25]), AD (HR = 1.04 [CI: 0.84–1.29]), or VaD (HR = 1.37 [CI: 0.81–2.34]), but associated with increased risk of mortality or dementia (composite) (HR = 1.51 [CI: 1.38–1.65]), over 9 years. Incident PAD not associated with risk of dementia, AD, or VaD.	Age, sex, SBP^d^, DBP^d^, DM^d^, stroke^d^	Dem^b^

a Only confounders considered in the quality assessment are listed.

b Prevalent at baseline.

c Same across cohort.

d Reported further adjustment for factors did not affect the pattern of results.

e Incident during follow up.

ABI, ankle–brachial index; AD, Alzheimer’s disease; CADDTC, California Alzheimer’s disease diagnostic and treatment centers; CAMDEX, Cambridge Mental Disorders of the Elderly Examination; CASI, cognitive abilities screening instrument; CERAD-K, Korean version of the consortium to establish a registry for an Alzheimer’s disease clinical assessment battery; CVD, cardiovascular disease; DBP, diastolic blood pressure; Dem, dementia (clinical diagnosis or MMSE scores < thresholds); DM, diabetes mellitus; DQ, Dementia Questionnaire; DSI, dementia screening indicator; DSM-III-R/-IV, *Diagnostic and Statistical Manual of Mental Disorders* (3rd Edition, 4th Edition); Edu, education; FSRP, Framingham Stroke Risk Profile; GMS-A, Geriatric Mental State Examination; HR, hazard ratio; HTN, hypertension; IQCODE, Informant Questionnaire on Cognitive Decline in the Elderly; IWG-MCI, International Working Group on Mild Cognitive Impairment; M [SD], mean [SD]; MCI, mild cognitive impairment; MMSE, Mini-Mental State Examination; 3MSE, Modified Mini-Mental State; N/A, not applicable; NIA-AA, National Institute on Aging–Alzheimer’s Association; NINCDS-ADRDA, National Institute of Neurological and Communication Disorders and Stroke–Alzheimer’s Disease and Related Disorders Association; NINDS-AIREN, National Institute of Neurological Disorders and Stroke–Association Internationale pour la Recherché et l’Enseignement en Neurosciences; NPB, neuropsychological battery; OR, odds ratio; PAD, peripheral artery disease; PY, person-years; SBP, systolic blood pressure; Smk, smoking; VaD, vascular dementia; WHO ICD-9/10, World Health Organization *International Classification of Diseases* (9th and 10th revisions).

Fifteen studies examined dementia risk with diagnoses of dementia made using validated criteria,^42,45,47,48,50,54,56,58,60,62,63,67,68,72^ or record linkage with national registers using the World Health Organization International Classification of Diseases (WHO ICD) codes.^53^ The most commonly used criteria were from the *Diagnostic and Statistical Manual of Mental Disorders, 3rd/4th Editions* (DSM-III/IV).^45,47,50,54,58,60,63,67,68,72^

### Quality assessment

Risk of bias assessments measured by the ROBINS-E tool are presented in Table S3. There was a high risk of bias in 34 studies and moderate risk of bias in four studies.^49,50,52,74^ The sources contributing to the high risk of bias in the majority of studies were due to confounding and missing data. Risk of bias due to confounding was high in 26 studies, moderate in two studies,^52,74^ and low in 10 studies.^35,40,49–51,57,58,62,66,69^ Risk of bias due to missing data was high in 22 studies, moderate in seven studies,^41,44,52,54,61,71,72^ and low in nine studies.^49,50,53,59,64,67,70,73,74^

### Cross-sectional association between PAD and cognitive function and impairment

#### Neuropsychological test batteries

Nine studies assessed various cognitive functions using neuropsychological test batteries;^39,40,48,49,57,59,61,64,66^ one assessed processing speed only.^51^ Results are presented in Table 1. All-but-one reported significant associations between PAD and poorer cognitive function in at least one domain.^40,48,49,51,57,59,61,64,66^ Two studies reported significantly worse performance in patients with PAD compared to age–education matched controls across several cognitive domains^59,64^ and one reported an association between low ABI and poorer memory scores only, in age–education adjusted analyses.^61^

Analyses in five studies were well-adjusted,^40,49,51,57,66^ and one adjusted for all factors except stroke.^48^ Three studies reported associations between PAD and poorer global cognition,^48,57^ processing speed,^48,57^ short and long-term memory,^48,49^ working memory,^49^ cognitive flexibility, and/or inhibition^48,57^ in fully adjusted models. Bareiro et al.^40^ reported an association between declining ABI and lower MMSE scores in the < 0.8 category, and worse short-term memory and visuospatial ability in each ABI category (1.4 to > 1.1; < 1.1 to 0.8; < 0.8) in fully adjusted models, with greater declines observed with ABI < 0.08.^40^ Tarraf et al.^66^ found an inverse u-shape association between ABI and global cognition, short and long-term memory, verbal fluency, and processing speed, with both low and high ABI values related to worse cognitive function. Associations were attenuated but remained significant in fully adjusted models.^66^

Cross-sectional evidence supports an association between PAD and cognitive function with all except one study^39^ reporting significant associations of PAD with poorer cognitive performance. In addition, associations were reported independent of age, education, smoking, hypertension, diabetes, and stroke in four of five studies adjusting for these factors.^40,49,57,66^ The specific cognitive functions assessed and results by separate domains for all studies are presented in Table S4. Impaired memory and processing speed are frequently observed in patients with PAD, with seven of nine studies reporting significant associations between PAD and poorer memory performance^40,48,49,59,61,64,66^ and/or processing speed.^48,49,51,57,59,64,66^ Associations remained significant in four^40,48,49,66^ and three^48,57,66^ of five studies that adjusted analyses for several factors. Aspects of executive function may also be impaired in patients with PAD, with a few studies reporting poorer performance independent of various confounders.^48,49,57^

#### Global cognitive function screening tests

Eleven studies assessed global cognitive function using screening assessments,^35,41,43,44,46,47,69–71,73,74^ and eight studies reported significant associations between PAD and worse global cognition or impairment^35,41,43,47,69–71,74^ (Table 1).

Analyses in seven studies were unadjusted,^44,46,71,73^ scores corrected for age and education,^43,47^ or adjusted for a few factors;^41^ four reported significant associations between PAD and impaired cognitive function (MMSE < 24/< 26),^41^ lower MMSE scores,^71^ single nonmemory cognitive impairment no dementia,^47^ and MCI (MMSE ⩽ 24).^43^ Analyses in three studies were well-adjusted and one adjusted for various confounders;^70^ all reported significant associations between cognitive impairment and low ABI (odds ratio [OR] = 1.58 [CI: 1.19–2.09]),^70^ low ABI and increased risk of cognitive impairment (OR = 1.98 [CI: 1.15–3.42])^35^ and subtle cognitive decline (OR = 2.96 [CI: 1.87–6.46]),^74^ and decreasing ABI and higher prevalence of MCI (prevalence ratio [PR] = 1.06 [CI: 1.01–1.11]) and nonamnestic MCI (PR = 1.12 [CI: 1.03–1.21]).^69^

The majority of studies reported significant associations between PAD and worse global cognitive function or impairment, half of which reported associations independent of various confounders.^35,69,70,74^ Effect estimates varied considerably though from approximately 50% to a threefold increased risk of cognitive impairment in those with an ABI < 0.9.^35,70,74^ In addition, all four studies assessing global cognition as part of a neuropsychological test battery reported significant associations between PAD and poorer performance independent of several factors.^40,48,57,66^

### Cross-sectional association between PAD and dementia

Six studies examined the cross-sectional association between PAD and dementia,^42,45,50,54,67,72^ five of which reported significant associations.^45,50,54,67,72^ Results are presented in Table 2. Analyses in two studies were unadjusted,^42,72^ one adjusted for age and sex,^54^ and two reported significant associations: one reported lower ABI values in individuals with dementia compared to those with normal cognition.^72^ In the Rotterdam study, PAD was associated with vascular dementia (VaD) (OR = 2.5 [CI: 1.3–4.8]) and all dementia (OR = 1.5 [CI: 1.1–2.0]), but not AD or other dementias.^54^

Three studies reported significant associations of PAD with dementia after adjustment for various factors including age, education,^45,50,67^ diabetes,^50,67^ hypertension,^50^ smoking,^45,50^ and stroke.^50^ Tasci et al.^67^ found an association between dementia and higher PAD prevalence (OR = 2.41 [CI: 1.34–4.32]).^67^ Two studies reported significant associations between low ABI and prevalent dementia (OR = 2.31 [CI: 1.28–4.17])^50^ and increased risk of cognitive impairment (MCI or dementia) (OR: 1.52 [CI: 1.03–2.25]).^45^

Cross-sectional evidence indicates an association of PAD and dementia, with all except one study^42^ reporting significant associations between PAD and dementia risk. However, all studies included participants with prevalent stroke, with only one study adjusting analyses for this factor.^50^ This makes it difficult to determine the independent effect of PAD on dementia development risk due to the strong association between cerebrovascular disease and cognitive impairment. Associations were reported independent of various cardiovascular and dementia development risk factors in half of studies,^45,50,67^ and cerebrovascular disease in one study adjusting for this factor.^50^

### Longitudinal association between PAD and cognitive function and impairment

#### Neuropsychological test batteries

Six studies assessed various cognitive domains using neuropsychological test batteries,^36,40,48,52,55,65^ and four reported significant associations of PAD with impairment in at least one cognitive domain.^36,40,52,55^ Results are presented in Table 3. Follow-up durations ranged from 2 to 15 years. Three studies adjusted analyses for a few factors,^36,55,65^ and two reported significant associations between lower baseline ABI and worse verbal fluency, processing speed, abstract reasoning,^36^ and composite global scores^55^ at 10 years. In fully adjusted models, associations remained significant for processing speed^36^ and global functioning.^55^ Baseline ABI or change in ABI (years 5–12) were not associated with change in cognitive function between years 10 and 15.^55^

Espeland et al.^48^ reported no association between baseline or 2-year change in ABI and 2-year change in cognitive function in analyses adjusted for all factors except stroke.^48^ Analyses in two studies were well-adjusted,^40,52^ one reported associations between baseline ABI and greater declines in short and long-term memory and executive function at 5 years in < 1.1 and > 1.1–1.4 ABI categories, with greater declines observed for ABI < 1.1.^40^ In the Cardiovascular Health Study (CHS), individuals with an ABI < 0.90 showed a greater decline in global cognition (MMSE) and processing speed over 7 years compared to those with an ABI of ⩾ 0.90.^52^

Evidence of the longitudinal association between PAD and impaired cognitive function is limited compared to cross-sectional evidence due to fewer published studies and a smaller proportion of studies reporting significant results. However, available evidence indicates that PAD is likely associated with impaired memory, processing speed, and executive function. Of the four studies assessing memory and/or processing speed, significantly worse performance or greater decline were reported in one^40^ and two studies,^36,52^ respectively, independent of various factors (Table S4). Two studies used composite executive function measures,^40,65^ one of which reported an association of PAD with executive dysfunction in well-adjusted analyses.^40^ This pattern of impairments is similar to those observed in cross-sectional studies, supporting the inference that particular areas of cognition may be impaired in patients with PAD.

#### Global cognitive function screening tests

Four studies assessed global cognition as part of a neuropsychological battery,^40,48,52,55^ with two reporting associations between PAD and significantly worse performance,^55^ or greater decline,^52^ independent of various factors. Evidence of the longitudinal association between PAD and global cognitive function and impairment is limited due to the small number of published studies; however, available evidence indicates that patients with PAD may have global cognitive impairments.

### Longitudinal association between PAD and dementia

Nine studies examined the longitudinal association between PAD and dementia,^47,48,53,56,58,60,62,63,68^ and four reported significant associations.^48,56,58,62^ Results are presented in Table 4. Analyses in three studies were unadjusted: none reported significant differences in baseline ABI between those who developed dementia over 3 years^47^ or 18 years,^53^ or progressed to MCI or dementia over 5 years,^60^ compared to those who did not. In age-adjusted analyses, Kuller et al.^56^ reported an association between low ABI and increased dementia incidence over 14 years in White women.

Five studies adjusted analyses for various factors including age, hypertension, or blood pressure, diabetes,^48,58,62,63,68^ education,^48,58,62,63^ smoking,^48,58,62,63^ and stroke,^58,62,68^ with three studies reporting associations with increased dementia risk.^48,58,62^ In the Rotterdam^68^ and Framingham Heart^63^ cohort studies, low baseline ABI was not associated with risk of incident dementia,^63,68^ AD,^63,68^ or VaD^68^ over 9–10 years. However, low ABI was associated with an increased risk of mortality or dementia (composite outcome measure) (hazard ratio [HR] = 1.51 [CI: 1.38–1.65]).^68^ Espeland et al.^48^ reported that lower baseline ABI was associated with increased risk of poorer cognitive outcome (progressing from normal cognition to MCI, or from normal cognition or MCI to probable dementia) at 2 years (OR = 2.60 [CI: 1.06–6.37]).^48^ Similarly, in the Honolulu-Asia Aging study, low baseline ABI was associated with increased risk of dementia (HR:1.79 [CI: 1.27–2.52]), VaD (HR = 2.79 [CI: 1.38–5.62]), and AD (HR=1.60 [CI: 1.01–2.53]) over 5 years, which remained significant for dementia in fully adjusted models (HR = 1.62 [CI: 1.13–2.32]).^58^ PAD was also associated with increased risk of total dementia (HR = 2.4 [CI: 1.4-4.0]), AD (HR = 2.4 [CI: 1.4–4.2]), and AD (excluding VaD) (HR = 2.2 [CI: 1.1–4.5]) over 5 years in the CHS-Cognition study.^62^

There was weaker evidence of an association between PAD and dementia in longitudinal studies, with a smaller proportion of studies reporting significant results. However, three of five longitudinal studies reported associations independent of age, education, hypertension, diabetes, smoking,^48,58,62^ and stroke.^58,62^ Follow-up durations in these studies were shorter, between 2 and 5 years, compared to 9 and 10 years in studies reporting no association with increased dementia risk.^63,68^ Associations with PAD were stronger for vascular or mixed dementia; however, associations with AD were also observed, suggesting that PAD may increase the risk of both vascular and Alzheimer’s type dementia.

## Discussion

This narrative review provides evidence that PAD is associated with both impaired cognitive function and increased risk of dementia. This association remained present in studies that adjusted for important confounders such as age, education, CRFs, and previous stroke. Those with PAD appear to predominantly have impairments in global cognitive function, memory, and processing speed, consistent with both vascular and Alzheimer’s type pathologies. Impairments in executive function may also be related to PAD with significantly worse performance reported in a few studies independent of various factors. Though increased risk of both cognitive impairment and dementia was demonstrated in cross-sectional and longitudinal studies, there is greater evidence of cross-sectional association as proportionally fewer longitudinal studies reported a significant association of PAD with development of dementia during follow up. However, similar effect sizes were observed in cross-sectional and longitudinal studies, and a third of longitudinal studies reported associations of PAD with increased dementia risk independent of several confounders, including cerebrovascular disease. The strong association between PAD and mortality may explain the weaker relationship between PAD and dementia observed in longitudinal studies, with individuals with PAD more likely to have died prior to the development or identification of cognitive impairment or dementia.^68^ Analyses in longitudinal studies also tended to be better adjusted, which may also explain the smaller proportion of studies reporting significant findings.

The results of this systematic review confirm the results of previous smaller review articles that reported increased cognitive impairment and dementia risk in patients with PAD.^28,30,31^ However, none of the previously published reviews considered risk of bias or variables included in the statistical models of included studies in their synthesis of results. There are a number of pathophysiological mechanisms that may explain the observed association of PAD with cognitive impairment and dementia. Low ABI is an indicator of generalized systemic atherosclerotic disease and may correlate with cognitive function due to the shared risk factors and underlying pathogenic mechanisms of PAD and cognitive impairment. Decreases in ABI may therefore be related to cognitive decline, especially in older adults who often present with a number of shared risk factors for both vascular disease and cognitive impairment.^48^ It is therefore vital to consider the degree to which confounders and bias may have contributed to observed results. The association was still present in many studies fully adjusting for confounders, lending further weight to the observed association of PAD with impaired cognitive function and risk of dementia.

PAD prevalence increases with age.^75^ Advancing age is associated with reduced cerebral blood flow (CBF) that is further compromised by CRFs and atherosclerotic disease.^76,77^ Suboptimal CBF and chronic hypoperfusion may lead to neuronal dysfunction and death, resulting in cognitive impairment.^77,78^ Greater brain atrophy and higher prevalence of subclinical cerebrovascular disease such as white matter hyperintensities (WMH) are observed in aging and atherosclerotic disease and are associated with cognitive decline and dementia.^79–86^ Cerebral hypoperfusion caused by vascular dysfunction is thought to contribute to WMH.^87^ White matter lesions potentially lead to cognitive decline by disrupting connectivity between distributed neural networks.^79,88^ Whether the observed cognitive impairments in patients with PAD result from damage to cortical and subcortical brain areas and white matter lesions through pathological processes such as atherosclerosis and ischemia is unclear. Studies indicate increased cerebral atrophy and subclinical cerebrovascular disease such as WMH and silent brain infarcts in patients with PAD;^85,89,90^ however, evidence is limited. Further neuroimaging studies are needed to investigate subclinical cerebral damage in patients with PAD and the resulting impact on cognitive function.

Patients with PAD often have atherosclerosis in other vascular beds, such as the carotid, coronary, and cerebral arteries.^28,91^ The impaired cognitive function observed in patients with PAD may result from reduced cerebral perfusion due to atherosclerosis in the larger cerebral arteries.^49,92^ In addition, atherosclerotic lesions in the intracerebral or carotid arteries could be the source of micro-emboli that, in significant numbers, may cause cognitive impairment.^92,93^ Evidence also indicates generalized microvascular dysfunction and damage in patients with PAD.^94–97^ The cerebral microvasculature ensures the metabolic needs of the brain are met, washout of metabolic by-products, and formation and regulation of the blood–brain barrier. Microvascular health is therefore vital to normal neuronal function.^98^ Neurovascular coupling (NVC), a physiological mechanism that ensures rapid adjustment of cerebral blood flow to activated neurons, is critical for normal brain function.^99^ Microvascular damage and cerebromicrovascular dysfunction, such as impaired NVC responses, likely contribute to reduced resting CBF and are associated with cognitive impairment.^98–101^ Associations between impaired microvascular endothelial function, NVC responses, and cognitive function have been reported in older adults with PAD.^100^ This supports the view that generalized microvascular dysfunction and subsequent impaired NVC responses contribute to the observed cognitive impairment in patients with PAD.^100^ Further research examining mechanisms of CBF regulation using techniques such as functional magnetic resonance imaging (fMRI), near infrared spectroscopy (NIRS), and transcranial doppler (TCD) ultrasound during cognitive stimulation are needed to confirm this as a pathological mechanism for cognitive impairment in PAD.

The prevalence of PAD and dementia is increasing globally.^33,75^ Dementia is a public health priority and with few targeted treatments, prevention and reducing risk by early identification and treatment of modifiable risk factors are of increasing importance.^102^ Modifiable risk factors for atherosclerosis, that also increase risk of dementia, tend to be therapeutically undertreated in patients with PAD compared to those with coronary heart disease.^103^ PAD is also underdiagnosed and undertreated.^104^ Many individuals with PAD are asymptomatic: approximately 10% of patients present with typical claudication symptoms, 50% experience atypical symptoms, and 40% are asymptomatic.^104^ Clinicians who rely on claudication history alone to diagnose PAD potentially miss 85–90% of cases.^105^ ABI is an indicator of structural and functional vascular changes in the arteries and can be used to diagnose PAD in the absence of clinical symptoms.^91,106^ ABI is an accurate, practical, cost-effective, and patient-acceptable measure and its use in clinical practice may identify individuals with asymptomatic and untreated PAD, providing an opportunity for early intervention and treatment, potentially reducing cardiovascular risk.^26,107^ Given the observed association of PAD with cognitive impairment and dementia identified in this review, the prevention or slowing of cognitive decline may be an additional potential benefit of PAD screening through early identification, prompting lifestyle change and initiation of optimal medical therapy. International guidelines currently do not recommend PAD screening due to lack of evidence of clinical benefit.^27,108^ However, the potential benefit from PAD screening in clinical practice in reducing the risk of cognitive impairment is currently unknown. This may be particularly relevant in low- and middle-income countries where PAD and dementia prevalence is rapidly rising but access to primary and preventative healthcare is more limited.^75,109,110^ Pooling of individual participant data from population-based studies could provide further information on the potential of PAD screening in determining risk of cognitive impairment.

The results from this systematic review also suggest that screening for cognitive impairment in patients with PAD should be considered. Referring patients to memory clinics for cognitive assessment may assist in early diagnosis of cognitive impairment, potentially leading to strategies to delay or prevent further decline or dementia and to better management. Identification of cognitive impairment may also be important in the management of patients with PAD.^49^ Impairments in memory, processing speed, and executive function are associated with difficulties in paying attention, remembering, decision making, organization, and planning, and may hinder patients’ understanding of, and adherence to, medical therapy, lifestyle changes, and exercise therapies. Awareness of cognitive impairments in patients with PAD may lead to more effective communication strategies with patients, increasing understanding and improving treatment and outcomes.

The strengths of this review include its size and breadth and consideration of risk of bias and the statistical models used by the included studies. There are a number of limitations though. There was a high risk of bias in most of the included studies, predominantly due to missing data and confounding which may have led to an overestimation of effect size. Approximately half of the included studies reported associations without adjustment, or following adjustment for a few factors, potentially resulting in over-estimation of the association in studies reporting significant results. However, of the 28 studies reporting significant results, 16 (57%) reported associations independent of various confounders including age, hypertension, diabetes, stroke, education, and smoking, of which 11 controlled for all factors. Other risk factors associated with PAD and dementia development, such as depression, obesity, alcohol consumption, and hyperlipidemia,^32,33,111,112^ were not considered when assessing bias risk or synthesizing results. However, significant associations between PAD and cognitive impairment were reported in all 11 studies that adjusted for depression and/or alcohol, and 10 of 12 studies that controlled for obesity and/or hyperlipidemia, indicating that the observed association of PAD with cognitive impairment is not explained by these factors.

Most of the studies were prospective population-based cohorts that are susceptible to attrition bias which could lead to an underestimation of effect size. It is possible that individuals with fewer comorbidities and better cognitive function were more likely to complete follow up. Additionally, survival bias must be considered, with higher mortality observed in individuals with more severe CVD during follow up: thus, these individuals may have died before either the onset or identification of cognitive impairment or dementia. Furthermore, although the majority of studies were population-based cohorts, individuals who participate are likely to be of better health when compared to the general population, potentially leading to selection bias. There is also potential publication bias; i.e., studies with nonsignificant results are less likely to be published, particularly observational studies,^113^ potentially leading to an over-estimation of the association of PAD and cognitive impairment. The wide scope of the review led to methodological and statistical heterogeneity among included studies, and therefore meta-analyses of aggregated data were not possible. Specifically, the neuropsychological tests used, cognitive domains assessed, assessment timepoints, and follow-up durations varied considerably among included studies. Furthermore, analysis methods were highly disparate between studies, particularly in their adjustment for potential confounders. PAD was most frequently related to impaired memory and processing speed, with associations present in several studies independent of various confounders. However, the results do not indicate that other cognitive functions are not affected in PAD, but instead that certain domains are less studied and/or have been studied predominantly in analyses with high bias risk. Most studies examining cognitive impairment and dementia development used standardized assessment thresholds and diagnostic criteria. However, the diagnostic workup for dementia diagnoses varied, with some studies using a combination of neuropsychological, functional, and neurological assessments, medical history, neuroimaging, and consensus-based adjudication by a panel (neurologists, psychologists/psychiatrists, geriatricians), and others using only a few.

## Conclusion

Evidence indicates an association of PAD with increased risk of cognitive impairment and dementia. Awareness of the prevalence of cognitive impairment and its potential impact on adherence to treatment and engagement in a healthy lifestyle is important for clinicians treating patients with PAD. Further research is needed to determine whether screening for, and optimal management of, PAD has cognitive benefits.

## Supplemental Material

sj-docx-1-vmj-10.1177_1358863X251336736 – Supplemental material for Association of cognitive impairment and peripheral artery disease (PAD): A systematic review Supplemental material, sj-docx-1-vmj-10.1177_1358863X251336736 for Association of cognitive impairment and peripheral artery disease (PAD): A systematic review by Beth L Cheshire, Sarah J Messeder, Coral J Pepper, Lucy C Beishon, Rob D Sayers and John SM Houghton in Vascular Medicine

sj-pdf-2-vmj-10.1177_1358863X251336736 – Supplemental material for Association of cognitive impairment and peripheral artery disease (PAD): A systematic review Supplemental material, sj-pdf-2-vmj-10.1177_1358863X251336736 for Association of cognitive impairment and peripheral artery disease (PAD): A systematic review by Beth L Cheshire, Sarah J Messeder, Coral J Pepper, Lucy C Beishon, Rob D Sayers and John SM Houghton in Vascular Medicine
