# Supplementary material for: Association of cognitive impairment and peripheral artery disease (PAD): A systematic review
Source: Vasc Med. 2025 May 21;30(6):724–39. doi: 10.1177/1358863X251336736 (PMC12664932; doi:10.1177/1358863X251336736)
Supplement: sj-docx-1-vmj-10.1177_1358863X251336736 – Supplemental material for Association of cognitive impairment and peripheral artery disease (PAD): A systematic review [file sj-docx-1-vmj-10.1177_1358863X251336736.docx]

**Supplemental Appendix. Search Strategies**

**Ovid MEDLINE(R) ALL <1946 to January 13, 2023> (17^th^ January 2023)**

1 Aging/ 247904

2 Cognitive Aging/ 1044

3 Longevity/ 24908

4 exp Aged/ 3431235

5 Geriatrics/ 31230

6 Middle Aged/ 4704985

7 Geriatric Assessment/ 31874

8 Age Factors/ 471996

9 (old* adj (people* or person* or man or woman or men or women or patient*)).tw,kw. 447099

10 (old* adj (adult* or age* or male* or female*)).tw,kw. 390287

11 "older population".tw,kw. 6416

12 centarian*.tw. 3

13 centenarian*.tw. 2309

14 nonagenarian*.tw. 1655

15 octagenarian*.tw. 48

16 octogenarian*.tw. 3777

17 septuagenarian*.tw. 461

18 supercentenarian*.tw. 118

19 elder*.tw,kw. 301220

20 geriatri*.tw. 56579

21 (ageing or aging).ti,kw. 121643

22 aged.ti,kw. 79052

23 middle age*.tw,kw. 61324

24 or/1-23 6403870

25 cohort*.ti,kw. 163506

26 longitudinal*.ti,kw. 87579

27 prospective*.ti,kw. 162724

28 observational.ti,kw. 47585

29 ((ageing or aging) adj (study or research*)).tw,kw. 6301

30 exp Cohort Studies/ 2435343

31 ("follow up" adj2 study).ti,kw. 22124

32 (study adj registr*).tw,kw. 1668

33 or/25-32 2572539

34 ((leg* or limb* or peripheral* or foot or feet) adj3 isch?emi*).tw,kw. 18269

35 (("blood vessel*" or arter* or vascular or peripheral*) adj3 (occlus* or reocclus* or "re‐occlus*" or steno* or restenos* or obstruct* or lesio* or block* or harden* or stiffen* or obliter* or calcificat* or restrict* or narrow* or block* or insufficien* or sclerosis)).tw. 170608

36 ((limb* or leg* or foot or feet or "lower extremit*") adj3 (occlus* or reocclus* or "re‐occlus*" or steno* or restenos* or obstruct* or lesio* or block* or harden* or stiffen* or obliter* or calcificat* or restrict* or narrow* or block* or insufficien* or sclerosis)).tw. 12761

37 ((femor* or iliac or popliteal or fempop* or crural or poplite* or infrapopliteal or inguinal or femdist* or inguinal or infrainquinal or tibial) adj3 (occlus* or reocclus* or "re‐occlus*" or steno* or restenos* or obstruct* or lesio* or block* or harden* or stiffen* or obliter*)).tw. 12173

38 (peripheral adj3 ("arter* disease*" or "arter* disorder*" or arteriopath*)).tw,kw. 18241

39 (peripheral adj3 ("vascul* disorder*" or "vascul* disease*")).tw,kw. 11180

40 PVD.tw,kw. 3098

41 PAOD.tw,kw. 837

42 atherosclero*.tw. 168584

43 arteriosclero*.tw. 16399

44 (ulcer* adj2 (arter* or vascular*)).tw. 1011

45 claudicat*.tw. 11595

46 Peripheral Vascular Diseases/ 12903

47 Peripheral Arterial Disease/ 10789

48 Blue Toe Syndrome/ 113

49 Arterial Occlusive Diseases/ 28169

50 Arteriosclerosis/ 56768

51 Arteriolosclerosis/ 178

52 Arteriosclerosis Obliterans/ 4032

53 Atherosclerosis/ 43850

54 Intermittent Claudication/ 8372

55 Ischemia/ 54184

56 ABPI.tw,kw. 484

57 ABI.tw,kw. 8127

58 ("ankle-brachial" adj3 (index* or indices)).tw,kw. 6814

59 rose questionnaire*.tw,kw. 170

60 WHO questionnaire*.tw. 140

61 edinburgh questionnaire*.tw,kw. 23

62 (toe* adj3 pressure*).tw,kw. 564

63 Ankle Brachial Index/ 3925

64 or/34-63 480895

65 "word recall".mp. 853

66 ("7‐minute screen*" or "seven‐minute screen*").mp. 0

67 "abbreviated mental test*".mp. 347

68 ((cogniti* or memory or "mental stat*") adj5 (tool* or assess* or score* or index* or identif* or scale* or questionnaire* or survey* or indicator* or system* or test* or screen* or evaluat* or exam*)).tw,kw. 176994

69 "animal fluency test*".mp. 119

70 "deterioration cognitive observee".mp. 5

71 "cognistat".mp. 91

72 "repeatable battery for the assessment of neuropsychological status".mp. 750

73 "self‐administered gerocognitive exam*".mp. 0

74 ("self‐administered" adj5 "SAGE").mp. 0

75 "short and sweet screening instrument".mp. 1

76 "short blessed test*".mp. 56

77 "telephone interview of cognitive status modified".mp. 49

78 "trail making test*".mp. 4599

79 "verbal fluency categories".mp. 4

80 "WORLD test".mp. 96

81 "Hopkins verbal learning test*".mp. 489

82 "time and change test*".mp. 8

83 "syndrome kurztest".mp. 4

84 "inform* interview*".tw,kw. 5917

85 ("6 CIT" or 6CIT or "ADAS-cog" or AD8 or "Dem Tect" or DemTect or IQCODE or "mini-cog" or MMSE or 3MS or QCST or RDST or RBANS or RUDAS or "s-omc" or SPMSQ or "tics-m" or GPCOG or HVLT or 7MS or CIDS or SAPH or AMTS or MOCA).mp. 21051

86 "Mental Status and Dementia Tests"/ 2943

87 ("organic brain disease*" or "organic brain syndrome*").tw,kw. 1077

88 "benign senescent forgetfulness".mp. 18

89 (pick* adj2 disease).tw,kw. 3485

90 (jcd or cjd).tw,kw. 3208

91 huntington*.tw,kw. 20552

92 binswanger*.tw,kw. 599

93 korsako*.tw,kw. 1703

94 ("GDS 3" or "stage 3 GDS").mp. 27

95 dement*.tw,kw. 141536

96 alzheimer*.tw,kw. 181458

97 (lewy* adj2 bod*).tw,kw. 11172

98 (chronic adj2 cerebrovascular).tw,kw. 775

99 ("pre-clinical AD" or "preclinical AD").tw,kw. 1038

100 ("global deterioration scale" and "stage 3").tw,kw. 3

101 exp Dementia/ 199077

102 Wernicke Encephalopathy/ 1875

103 "Neurocognitive Disorders"/ 9710

104 "Cognition Disorders"/ 66425

105 or/65-104 513074

106 24 and 33 and 64 and 105 996

**Embase <1974 to 2023 January 13> (17^th^ January 2023)**

1 exp aging/ 306824

2 longevity/ 32860

3 exp aged/ 3502466

4 middle aged/ 1938718

5 exp geriatrics/ 40604

6 geriatric assessment/ 20285

7 geriatric care/ 15352

8 age/ 570991

9 "growth, development and aging"/ 195641

10 (old* adj (people* or person* or man or woman or men or women or patient*)).tw,kw. 606624

11 (old* adj (adult* or age* or male* or female*)).tw,kw. 582119

12 "older population".tw,kw. 9070

13 centarian*.tw. 3

14 centenarian*.tw. 2769

15 nonagenarian*.tw. 2357

16 octagenarian*.tw. 110

17 octogenarian*.tw. 6100

18 septuagenarian*.tw. 660

19 supercentenarian*.tw. 130

20 elder*.tw,kw. 428643

21 geriatri*.tw. 89551

22 (ageing or aging).ti,kw. 157897

23 aged.ti,kw. 90252

24 middle age*.tw,kw. 79006

25 or/1-24 6129798

26 cohort*.ti,kw. 233170

27 longitudinal*.ti,kw. 111901

28 prospective*.ti,kw. 227045

29 observational.ti,kw. 64462

30 ((ageing or aging) adj (study or research*)).tw,kw. 8073

31 ("follow up" adj2 study).ti,kw. 26080

32 (study adj registr*).tw,kw. 1501

33 cohort analysis/ 944830

34 exp longitudinal study/ 184662

35 prospective study/ 822501

36 retrospective study/ 1366874

37 or/26-36 2960805

38 ((leg* or limb* or peripheral* or foot or feet) adj3 isch?emi*).tw,kw. 27161

39 (("blood vessel*" or arter* or vascular or peripheral*) adj3 (occlus* or reocclus* or "re‐occlus*" or steno* or restenos* or obstruct* or lesio* or block* or harden* or stiffen* or obliter* or calcificat* or restrict* or narrow* or block* or insufficien* or sclerosis)).tw. 236670

40 ((limb* or leg* or foot or feet or "lower extremit*") adj3 (occlus* or reocclus* or "re‐occlus*" or steno* or restenos* or obstruct* or lesio* or block* or harden* or stiffen* or obliter* or calcificat* or restrict* or narrow* or block* or insufficien* or sclerosis)).tw. 17503

41 ((femor* or iliac or popliteal or fempop* or crural or poplite* or infrapopliteal or inguinal or femdist* or inguinal or infrainquinal or tibial) adj3 (occlus* or reocclus* or "re‐occlus*" or steno* or restenos* or obstruct* or lesio* or block* or harden* or stiffen* or obliter*)).tw. 17645

42 (peripheral adj3 ("arter* disease*" or "arter* disorder*" or arteriopath*)).tw,kw. 29029

43 (peripheral adj3 ("vascul* disorder*" or "vascul* disease*")).tw,kw. 16625

44 PVD.tw,kw. 5120

45 PAOD.tw,kw. 1403

46 atherosclero*.tw. 233930

47 arteriosclero*.tw. 16938

48 (ulcer* adj2 (arter* or vascular*)).tw. 1515

49 claudicat*.tw. 16043

50 peripheral vascular disease/ 24418

51 exp peripheral occlusive artery disease/ 193162

52 exp blood vessel calcification/ 26361

53 artery occlusion/ 28155

54 blood vessel occlusion/ 13502

55 exp arteriosclerosis/ 276367

56 exp limb ischemia/ 19868

57 ABPI.tw,kw. 794

58 ABI.tw,kw. 16183

59 ("ankle-brachial" adj3 (index* or indices)).tw,kw. 10407

60 rose questionnaire*.tw,kw. 236

61 WHO questionnaire*.tw. 189

62 edinburgh questionnaire*.tw,kw. 37

63 (toe* adj3 pressure*).tw,kw. 781

64 ankle brachial index/ 13351

65 or/38-64 742152

66 "word recall".mp. 1199

67 ("7‐minute screen*" or "seven‐minute screen*").mp. 0

68 "abbreviated mental test*".mp. 649

69 ((cogniti* or memory or "mental stat*") adj5 (tool* or assess* or score* or index* or identif* or scale* or questionnaire* or survey* or indicator* or system* or test* or screen* or evaluat* or exam*)).tw,kw. 251113

70 "animal fluency test*".mp. 190

71 "deterioration cognitive observee".mp. 6

72 "cognistat".mp. 153

73 "repeatable battery for the assessment of neuropsychological status".mp. 1207

74 "self‐administered gerocognitive exam*".mp. 1

75 ("self‐administered" adj5 "SAGE").mp. 1

76 "short and sweet screening instrument".mp. 1

77 "short blessed test*".mp. 101

78 "telephone interview of cognitive status modified".mp. 92

79 "trail making test*".mp. 8878

80 "verbal fluency categories".mp. 5

81 "WORLD test".mp. 105

82 "Hopkins verbal learning test*".mp. 1393

83 "time and change test*".mp. 11

84 "syndrome kurztest".mp. 5

85 "inform* interview*".tw,kw. 6990

86 ("6 CIT" or 6CIT or "ADAS-cog" or AD8 or "Dem Tect" or DemTect or IQCODE or "mini-cog" or MMSE or 3MS or QCST or RDST or RBANS or RUDAS or "s-omc" or SPMSQ or "tics-m" or GPCOG or HVLT or 7MS or CIDS or SAPH or AMTS or MOCA).mp. 42485

87 exp dementia assessment/ 63350

88 ("organic brain disease*" or "organic brain syndrome*").tw,kw. 1374

89 "benign senescent forgetfulness".mp. 31

90 (pick* adj2 disease).tw,kw. 4353

91 (jcd or cjd).tw,kw. 4715

92 huntington*.tw,kw. 27975

93 binswanger*.tw,kw. 787

94 korsako*.tw,kw. 1888

95 ("GDS 3" or "stage 3 GDS").mp. 60

96 dement*.tw,kw. 205213

97 alzheimer*.tw,kw. 248312

98 (lewy* adj2 bod*).tw,kw. 17005

99 (chronic adj2 cerebrovascular).tw,kw. 1224

100 ("pre-clinical AD" or "preclinical AD").tw,kw. 1939

101 ("global deterioration scale" and "stage 3").tw,kw. 18

102 exp dementia/ 423994

103 Wernicke encephalopathy/ 3050

104 "disorders of higher cerebral function"/ 2868

105 cognitive defect/ 203342

106 mild cognitive impairment/ 34709

107 memory disorder/ 42625

108 or/66-107 842286

109 25 and 37 and 65 and 108 2220

**PsycInfo (including PsycArticles) strategy 17^th^ January 2023**

**903 results**

(MAINSUBJECT.EXACT("Gerontology") OR MAINSUBJECT.EXACT.EXPLODE("Aging") OR MAINSUBJECT.EXACT("Aged (Attitudes Toward)") OR MAINSUBJECT.EXACT("Geriatrics") OR MAINSUBJECT.EXACT("Cognitive Aging") OR MAINSUBJECT.EXACT("Middle Adulthood") OR MAINSUBJECT.EXACT("Geriatric Assessment") OR MAINSUBJECT.EXACT("Age Differences") OR noft(old* NEAR/1 (people* OR person* OR man OR woman OR men OR women OR patient*)) OR noft(old* NEAR/1 (adult* OR age* OR male* OR female*)) OR noft("older population") OR noft(centarian* OR centenarian* OR nonagenarian* OR octagenarian* OR octogenarian* OR septuagenarian* OR supercentenarian*) OR noft(elder* OR geriatri* OR ageing OR aging OR aged OR "middle age*"))

AND (noft(cohort* OR longitudinal* OR prospective* OR observational) OR noft((ageing OR aging) NEAR/1 (study OR research*)) OR noft("follow up" NEAR/2 study) OR noft(study NEAR/1 registr*) OR MAINSUBJECT.EXACT("Cohort Analysis") OR MAINSUBJECT.EXACT.EXPLODE("Longitudinal Studies"))

AND (noft(ulcer* NEAR/2 (arter* OR vascular*)) OR noft(claudicat*) OR MAINSUBJECT.EXACT("Arteriosclerosis") OR MAINSUBJECT.EXACT("Atherosclerosis") OR MAINSUBJECT.EXACT("Ischemia") OR MAINSUBJECT.EXACT("Cardiovascular Disorders") OR noft(ABPI OR ABI) OR noft("ankle-brachial" NEAR/3 (index* OR indices)) OR noft("rose questionnaire*" OR "WHO questionnaire*" OR "edinburgh questionnaire*") OR noft(toe* NEAR/3 pressure*) OR noft((leg* OR limb* OR peripheral* OR foot OR feet) NEAR/3 isch?emi*) OR noft(peripheral NEAR/3 ("arter* disease*" OR "arter* disorder*" OR arteriopath*)) OR noft(peripheral NEAR/3 ("vascul* disorder*" OR "vascul* disease*")) OR noft(PVD) OR noft(PAOD) OR noft(atherosclero*) OR noft(arteriosclero*))

AND (("7‐minute screen*" OR "seven‐minute screen*" OR "abbreviated mental test*") OR noft((cogniti* OR memory OR "mental stat*") NEAR/5 (tool* OR assess* OR score* OR index* OR identif* OR scale* OR questionnaire* OR survey* OR indicator* OR system* OR test* OR screen* OR evaluat* OR exam*)) OR "animal fluency test*" OR "deterioration cognitive observee" OR "cognistat" OR "repeatable battery for the assessment of neuropsychological status" OR "self‐administered gerocognitive exam*" OR ("self‐administered" NEAR/5 "SAGE") OR "short and sweet screening instrument" OR "short blessed test*" OR "telephone interview of cognitive status modified" OR "trail making test*" OR "verbal fluency categories" OR "WORLD test" OR "Hopkins verbal learning test*" OR "time and change test*" OR "syndrome kurztest" OR noft("inform* interview*") OR ("6 CIT" OR 6CIT OR "ADAS-cog" OR AD8 OR "Dem Tect" OR DemTect OR IQCODE OR "mini-cog" OR MMSE OR 3MS OR QCST OR RDST OR RBANS OR RUDAS OR "s-omc" OR SPMSQ OR "tics-m" OR GPCOG OR HVLT OR 7MS OR CIDS OR SAPH OR AMTS OR MOCA) OR MAINSUBJECT.EXACT("Cognitive Assessment") OR noft("organic brain disease*" OR "organic brain syndrome*") OR "benign senescent forgetfulness" OR noft(pick* NEAR/2 disease) OR noft(jcd OR cjd OR huntington* OR binswanger* OR korsako* OR dement* OR alzheimer*) OR "GDS 3" OR "stage 3 GDS" OR noft(lewy* NEAR/2 bod*) OR noft(chronic NEAR/2 cerebrovascular) OR noft("pre-clinical AD" OR "preclinical AD") OR noft("global deterioration scale" AND "stage 3") OR MAINSUBJECT.EXACT.EXPLODE("Dementia") OR MAINSUBJECT.EXACT("Wernicke's Syndrome") OR MAINSUBJECT.EXACT("Memory Disorders") OR MAINSUBJECT.EXACT("Neurocognitive Disorders") OR MAINSUBJECT.EXACT("Cognitive Impairment"))

**CENTRAL – 17^th^ January 2023**

ID Search Hits

#1 MeSH descriptor: [Aging] explode all trees 3940

#2 MeSH descriptor: [Aged] explode all trees 221613

#3 MeSH descriptor: [Geriatrics] this term only 213

#4 MeSH descriptor: [Middle Aged] this term only 334475

#5 MeSH descriptor: [Geriatric Assessment] this term only 1595

#6 MeSH descriptor: [Age Factors] explode all trees 10399

#7 (old* NEXT (people* or person* or man or woman or men or women or patient*)) 19440

#8 old* NEXT (adult* or age* or male* or female*) 25977

#9 "older population" 736

#10 (centarian* OR centenarian* OR nonagenarian* OR octagenarian* OR octogenarian* OR septuagenarian* OR supercentenarian*):ti,ab 178

#11 elder* 60373

#12 geriatri*:ti,ab 7061

#13 (ageing OR aging):ti,kw 10405

#14 aged:ti,kw 471209

#15 middle NEXT age* 376949

#16 {OR #1-#15} 529976

#17 cohort* 74129

#18 longitudinal* 26211

#19 prospective* 272108

#20 observational 25103

#21 (ageing or aging) NEXT (study or research*) 662

#22 ("follow up" NEAR/2 study) 25667

#23 study NEXT registr* 628

#24 MeSH descriptor: [Cohort Studies] explode all trees 161915

#25 {OR #17-#24} 414371

#26 (leg* or limb* or peripheral* or foot or feet) NEAR/3 isch?emi* 2936

#27 ((blood NEXT vessel*) or arter* or vascular or peripheral*) NEAR/3 (occlus* or reocclus* or (re NEXT occlus*) or steno* or restenos* or obstruct* or lesio* or block* or harden* or stiffen* or obliter* or calcificat* or restrict* or narrow* or block* or insufficien* or sclerosis) 18976

#28 (limb* or leg* or foot or feet or (lower NEXT extremit*)) NEAR/3 (occlus* or reocclus* or (re NEXT occlus*) or steno* or restenos* or obstruct* or lesio* or block* or harden* or stiffen* or obliter* or calcificat* or restrict* or narrow* or block* or insufficien* or sclerosis) 2219

#29 (femor* or iliac or popliteal or fempop* or crural or poplite* or infrapopliteal or inguinal or femdist* or inguinal or infrainquinal or tibial) NEAR/3 (occlus* or reocclus* or (re NEXT occlus*) or steno* or restenos* or obstruct* or lesio* or block* or harden* or stiffen* or obliter*) 3733

#30 peripheral NEAR/3 ((arter* NEXT (disease* OR disorder*)) or arteriopath*) 4532

#31 peripheral NEAR/3 (vascul* NEXT (disorder* or disease*)) 2740

#32 PVD 16121

#33 PAOD 246

#34 atherosclero* 15737

#35 arteriosclero* 3102

#36 ulcer* NEAR/2 (arter* or vascular*) 228

#37 claudicat* 2944

#38 MeSH descriptor: [Peripheral Vascular Diseases] this term only 941

#39 MeSH descriptor: [Peripheral Arterial Disease] this term only 1254

#40 MeSH descriptor: [Blue Toe Syndrome] this term only 1

#41 MeSH descriptor: [Arterial Occlusive Diseases] this term only 945

#42 MeSH descriptor: [Arteriosclerosis] this term only 1033

#43 MeSH descriptor: [Arteriolosclerosis] this term only 0

#44 MeSH descriptor: [Arteriosclerosis Obliterans] this term only 89

#45 MeSH descriptor: [Atherosclerosis] this term only 1551

#46 MeSH descriptor: [Intermittent Claudication] this term only 995

#47 MeSH descriptor: [Ischemia] this term only 2385

#48 ABPI 199

#49 ABI 1798

#50 "ankle-brachial" NEAR/3 (index* or indices) 1764

#51 rose NEXT questionnaire* 21

#52 WHO NEXT questionnaire* 18

#53 edinburgh NEXT questionnaire* 27

#54 toe* NEAR/3 pressure* 157

#55 MeSH descriptor: [Ankle Brachial Index] this term only 240

#56 {OR #26-#55} 57844

#57 "word recall" 276

#58 ("7‐minute" OR "seven-minute") NEXT screen* 16

#59 "abbreviated mental" NEXT test* 121

#60 (cogniti* or memory or (mental NEXT stat*)) NEAR/5 (tool* or assess* or score* or index* or identif* or scale* or questionnaire* or survey* or indicator* or system* or test* or screen* or evaluat* or exam*) 43710

#61 "animal fluency" NEXT test* 24

#62 "deterioration cognitive observee" 15

#63 "cognistat" 24

#64 "repeatable battery for the assessment of neuropsychological status" 251

#65 "self‐administered gerocognitive" NEXT exam* 21

#66 "self‐administered" NEAR/5 SAGE 20

#67 "short and sweet screening instrument" 12

#68 "short blessed" NEXT test* 26

#69 "telephone interview of cognitive status modified" 17

#70 "trail making" NEXT test* 1767

#71 "verbal fluency categories" 14

#72 "WORLD test" 18

#73 "Hopkins verbal learning" NEXT test* 356

#74 "time and change" NEXT test* 12

#75 "syndrome kurztest" 17

#76 inform* NEXT interview* 354

#77 ("6 CIT" or 6CIT or "ADAS-cog" or AD8 or "Dem Tect" or DemTect or IQCODE or "mini-cog" or MMSE or 3MS or QCST or RDST or RBANS or RUDAS or "s-omc" or SPMSQ or "tics-m" or GPCOG or HVLT or 7MS or CIDS or SAPH or AMTS or MOCA) 8888

#78 MeSH descriptor: [Mental Status and Dementia Tests] explode all trees 646

#79 "organic brain" NEXT (disease* or syndrome*) 260

#80 "benign senescent forgetfulness" 81

#81 pick* NEAR/2 disease 203

#82 jcd or cjd 218

#83 huntington* 1278

#84 binswanger* 116

#85 korsako* 807

#86 ("GDS 3" or "stage 3 GDS") 42

#87 dement* 29056

#88 alzheimer* 14468

#89 lewy* NEAR/2 bod* 620

#90 chronic NEAR/2 cerebrovascular 819

#91 "pre-clinical AD" OR "preclinical AD" 101

#92 "global deterioration scale" and "stage 3" 27

#93 MeSH descriptor: [Dementia] explode all trees 6885

#94 MeSH descriptor: [Wernicke Encephalopathy] this term only 5

#95 MeSH descriptor: [Neurocognitive Disorders] this term only 212

#96 MeSH descriptor: [Cognition Disorders] this term only 3600

#97 {OR #57-#96} 69708

#98 #16 AND #25 AND #56 AND #97 361 (248 from CENTRAL)

**CINAHL – 17^th^ January 2023**

S1 (MH "Aging+") 61,714

S2 (MH "Aged+") 936,449

S3 (MH "Geriatrics") 6,018

S4 (MH "Middle Age") 1,126,953

S5 (MH "Geriatric Assessment+") 18,449

S6 (MH "Age Factors") 144,727

S7 TI ( (old* N1 (people* or person* or man or woman or men or women or patient*)) ) OR AB ( (old* N1 (people* or person* or man or woman or men or women or patient*)) ) 93,205

S8 TI ( (old* N1 (adult* or age* or male* or female*)) ) OR AB ( (old* N1 (adult* or age* or male* or female*)) ) 127,464

S9 TI "older population" OR AB "older population" 3,081

S10 TI centarian* OR AB centarian* 2

S11 TI centenarian* OR AB centenarian* 810

S12 TI nonagenarian* OR AB nonagenarian* 674

S13 TI octagenarian* OR AB octagenarian* 14

S14 TI octogenarian* OR AB octogenarian* 1,205

S15 TI septuagenarian* OR AB septuagenarian* 135

S16 TI supercentenarian* OR AB supercentenarian* 67

S17 TI elder* OR AB elder* 114,210

S18 TI geriatri* OR AB geriatri* 29,289

S19 TI ( (ageing OR aging) ) OR AB ( (ageing OR aging) ) 74,614

S20 TI aged OR AB aged 232,467

S21 TI middle N1 age* OR AB middle N1 age* 19,914

S22 S1 OR S2 OR S3 OR S4 OR S5 OR S6 OR S7 OR S8 OR S9 OR S10 OR S11 OR S12 OR S13 OR S14 OR S15 OR S16 OR S17 OR S18 OR S19 OR S20 OR S21 1,735,868

S23 TI cohort* OR AB cohort* 290,054

S24 TI longitudinal* OR AB longitudinal* 115,574

S25 TI prospective* OR AB prospective* 259,659

S26 TI observational OR AB observational 93,700

S27 TI ( (ageing or aging) N1 (study or research*) ) OR AB ( (ageing or aging) N1 (study or research*) ) 7,760

S28 TI "follow up" N2 study OR AB "follow up" N2 study 22,004

S29 TI study N1 registr* OR AB study N1 registr* 5,820

S30 (MH "Prospective Studies+") 517,518

S31 S23 OR S24 OR S25 OR S26 OR S27 OR S28 OR S29 OR S30 848,165

S32 TI ( ((leg* or limb* or peripheral* or foot or feet) N3 isch?emi*) ) OR AB ( ((leg* or limb* or peripheral* or foot or feet) N3 isch?emi*) ) 543

S33 TI ( (("blood vessel*" or arter* or vascular or peripheral*) N3 (occlus* or reocclus* or "re‐ occlus*" or steno* or restenos* or obstruct* or lesio* or block* or harden* or stiffen* or obliter* or calcificat* or restrict* or narrow* or block* or insufficien* or sclerosis)) ) OR AB ( (("blood vessel*" or arter* or vascular or peripheral*) N3 (occlus* or reocclus* or "re‐ occlus*" or steno* or restenos* or obstruct* or lesio* or block* or harden* or stiffen* or obliter* or calcificat* or restrict* or narrow* or block* or insufficien* or sclerosis)) ) 31,326

S34 TI ( ((limb* or leg* or foot or feet or "lower extremit*") N3 (occlus* or reocclus* or "re‐occlus*" or steno* or restenos* or obstruct* or lesio* or block* or harden* or stiffen* or obliter* or calcificat* or restrict* or narrow* or block* or insufficien* or sclerosis)) ) OR AB ( ((limb* or leg* or foot or feet or "lower extremit*") N3 (occlus* or reocclus* or "re‐occlus*" or steno* or restenos* or obstruct* or lesio* or block* or harden* or stiffen* or obliter* or calcificat* or restrict* or narrow* or block* or insufficien* or sclerosis)) ) 3,639

S35 TI ( ((femor* or iliac or popliteal or fempop* or crural or poplite* or infrapopliteal or inguinal or femdist* or inguinal or infrainquinal or tibial) N3 (occlus* or reocclus* or "re‐occlus*" or steno* or restenos* or obstruct* or lesio* or block* or harden* or stiffen* or obliter*)) ) OR AB ( ((femor* or iliac or popliteal or fempop* or crural or poplite* or infrapopliteal or inguinal or femdist* or inguinal or infrainquinal or tibial) N3 (occlus* or reocclus* or "re‐occlus*" or steno* or restenos* or obstruct* or lesio* or block* or harden* or stiffen* or obliter*)) ) 3,424

S36 TI ( (peripheral N3 ("arter* disease*" or "arter* disorder*" or arteriopath*)) ) OR AB ( (peripheral N3 ("arter* disease*" or "arter* disorder*" or arteriopath*)) ) 5,468

S37 TI ( (peripheral N3 ("vascul* disorder*" or "vascul* disease*")) ) OR AB ( (peripheral N3 ("vascul* disorder*" or "vascul* disease*")) ) 2,524

S38 TI PVD OR AB PVD 564

S39 TI PAOD OR AB PAOD 126

S40 TI atherosclero* OR AB atherosclero* 28,643

S41 TI arteriosclero* OR AB arteriosclero* 961

S42 TI ( (ulcer* N2 (arter* or vascular*)) ) OR AB ( (ulcer* N2 (arter* or vascular*)) ) 520

S43 TI claudicat* OR AB claudicat* 2,291

S44 (MH "Peripheral Vascular Diseases+") 19,716

S45 (MH "Arterial Occlusive Diseases") 3,798

S46 (MH "Arteriosclerosis") 6,778

S47 (MH "Atherosclerosis") 10,911

S48 (MH "Intermittent Claudication") 1,548

S49 TI ABPI OR AB ABPI 227

S50 TI ABI OR AB ABI 2,556

S51 TI ( "ankle-brachial" N3 (index* or indices) ) OR AB ( "ankle-brachial" N3 (index* or indices) ) 2,077

S52 TI rose N1 questionnaire* OR AB rose N1 questionnaire* 68

S53 TI WHO N1 questionnaire* OR AB WHO N1 questionnaire* 724

S54 TI edinburgh N1 questionnaire* OR AB edinburgh N1 questionnaire* 80

S55 TI toe* N3 pressure* OR AB toe* N3 pressure* 184

S56 (MH "Ankle Brachial Index") 2,334

S57 S32 OR S33 OR S34 OR S35 OR S36 OR S37 OR S38 OR S39 OR S40 OR S41 OR S42 OR S43 OR S44 OR S45 OR S46 OR S47 OR S48 OR S49 OR S50 OR S51 OR S52 OR S53 OR S54 OR S55 OR S56 96,853

S58 "word recall" 306

S59 ("7‐minute screen*" or "seven‐minute screen*") 24

S60 "abbreviated mental test*" 203

S61 TI ( ((cogniti* or memory or "mental stat*") N5 (tool* or assess* or score* or index* or identif* or scale* or questionnaire* or survey* or indicator* or system* or test* or screen* or evaluat* or exam*)) ) OR AB ( ((cogniti* or memory or "mental stat*") N5 (tool* or assess* or score* or index* or identif* or scale* or questionnaire* or survey* or indicator* or system* or test* or screen* or evaluat* or exam*)) ) 65,877

S62 "animal fluency test*" 65

S63 "deterioration cognitive observee" 1

S64 cognistat 49

S65 "repeatable battery for the assessment of neuropsychological status" 249

S66 "self‐administered gerocognitive exam*" 6

S67 "self‐administered" N5 "SAGE" 5

S68 "short and sweet screening instrument" 0

S69 "short blessed test*" 25

S70 "telephone interview of cognitive status modified" 32

S71 "trail making test*" 1,556

S72 "verbal fluency categories" 3

S73 "WORLD test" 21

S74 "Hopkins verbal learning test*" 195

S75 "time and change test*" 6

S76 "syndrome kurztest" 0

S77 TI "inform* interview*" OR AB "inform* interview*" 3,450

S78 ("6 CIT" or 6CIT or "ADAS-cog" or AD8 or "Dem Tect" or DemTect or IQCODE or "mini-cog" or MMSE or 3MS or QCST or RDST or RBANS or RUDAS or "somc" or SPMSQ or "ticsm" or GPCOG or HVLT or 7MS or CIDS or SAPH or AMTS or MOCA) 10,015

S79 (MH "Short Portable Mental Status Questionnaire") 19,577

S80 (MH "Diagnosis, Psychosocial") 5,629

S81 TI ( ("organic brain disease*" or "organic brain syndrome*") ) OR AB ( ("organic brain disease*" or "organic brain syndrome*") )112

S82 "benign senescent forgetfulness" 3

S83 TI pick* N2 disease OR AB pick* N2 disease 411

S84 TI ( jcd or cjd ) OR AB ( jcd or cjd ) 550

S85 TI huntington* OR AB huntington* 2,530

S86 TI binswanger* OR AB binswanger* 58

S87 TI korsako* OR AB korsako* 230

S88 ("GDS 3" or "stage 3 GDS") 3

S89 TI dement* OR AB dement* 65,669

S90 TI alzheimer* OR AB alzheimer* 42,526

S91 TI lewy* N2 bod* OR AB lewy* N2 bod* 2,734

S92 TI chronic N2 cerebrovascular OR AB chronic N2 cerebrovascular 203

S93 TI ( ("pre-clinical AD" or "preclinical AD") ) OR AB ( ("pre-clinical AD" or "preclinical AD") ) 272

S94 ("global deterioration scale" and "stage 3") 3

S95 (MH "Dementia+") 84,087

S96 (MH "Wernicke's Encephalopathy") 476

S97 (MH "Cognition Disorders+") 36,365

S98 S58 OR S59 OR S60 OR S61 OR S62 OR S63 OR S64 OR S65 OR S66 OR S67 OR S68 OR S69 OR S70 OR S71 OR S72 OR S73 OR S74 OR S75 OR S76 OR S77 OR S78 OR S79 OR S80 OR S81 OR S82 OR S83 OR S84 OR S85 OR S86 OR S87 OR S88 OR S89 OR S90 OR S91 OR S92 OR S93 OR S94 OR S95 OR S96 OR S97 212,790

S99 S22 AND S31 AND S57 AND S98 600
