# Supplementary material for: Association of cognitive impairment and peripheral artery disease (PAD): A systematic review
Source: Vasc Med. 2025 May 21;30(6):724–39. doi: 10.1177/1358863X251336736 (PMC12664932; doi:10.1177/1358863X251336736)
Supplement: sj-pdf-2-vmj-10.1177_1358863X251336736 – Supplemental material for Association of cognitive impairment and peripheral artery disease (PAD): A systematic review [file sj-pdf-2-vmj-10.1177_1358863X251336736.pdf]

Table S1. Inclusion and exclusion criteria

Inclusion criteria	Exclusion criteria
1. Studies that assessed PAD defined by an ABI of ≤0.9 and;<br>2. Studies that assessed cognitive function, impairment or dementia (screening tool, cognitive assessment, clinical diagnosis) and;<br>3. Reported their association in adult populations.	1. Studies in which assessments of ABI and cognitive function were not performed in the same population of patients.<br>2. Studies in which participants with or without PAD were excluded or not cognitively assessed.<br>3. Studies in which the association between PAD and cognition was not reported separately.<br>4. Studies including disease-specific cohorts without use of healthy controls.<br>5. Duplicate publications from the same cohort and wave.

ABI= ankle brachial index; PAD= peripheral artery disease

Table S2. Main characteristics of the studies included in the review

Author	Country (study)	Design	Recruitment date	Follow up duration	N (% male)	Age range/ M(SD) (years)	PAD assessment	Proportion with PAD	Outcome
André-Petersson (2001)	Sweden (Men Born in 1914 study)	Prospective cohort*	1982-83	N/A	500 (100)	68-69	ABI <0.9	13.6%	Cognition†
Bareiro (2024)	Spain (TSHA)	Prospective cohort	2006-09	5 years	1147 (42.4)	≥65<br>74.3 (5.6)	ABI <0.8; 0.8 to <1.1; >1.1 to 1.4	5.4%	Cognition†
Breteler (1994)	Netherlands (Rotterdam Study)	Prospective cohort*	1990-92	N/A	4971 (NR)	55-94	ABI <0.9	21.0%	Cognitive impairment
Bruunsgaard (1999)	Denmark (Danish Centenarian)	Prospective cohort*	1995-96	N/A	126 (24.0)	100	ABI <0.9	55.2%	Dementia
Buscemi (2017)	Italy (ABCD study)	Prospective cohort*	2015	N/A	438 (NR)	≥55<br>MCI: 68 (6.0)<br>No MCI: 64 (6.0)	ABI <0.9	NR	Cognition†<br>Cognitive impairment
Chen (2018)	Taiwan	Cross-sectional	2015-16	N/A	985 (19.3)	≥60<br>70.8 (6.8)	ABI <0.9	3.0%	Cognition†
Chen (2024)	China (Tianning Cohort)	Prospective cohort*	2018	N/A	CN: 5082 (41.6)<br>CI: 76 (30.3)	18-96<br>CN: 50.7 (15.6)<br>CI: 65.0 (10.1)	ABI	NR	Cognitive impairment
Desormais (2018)	CAR, DRC (EPIDEMCA)	Prospective cohort*	2011-12	N/A	1662 (40.7)	≥65<br>72.9 (6.5)	ABI ≤0.9; 0.91 to 1.39; ≥1.40	14.3%	Dementia
DiCarlo (2000)	Italy (ILSA)	Prospective cohort*	1992-93	N/A	3425 (52.4)	65-84<br>74.4 (5.7)	ABI <0.8/ PMH	NR	Cognitive impairment
DiCarlo (2007)	Italy (ILSA)	Prospective cohort	1992-93	3 years	2,768 (53.6)	65-84<br>73.6 (5.5)	ABI <0.8/ PMH	7.8%	Cognitive impairment<br>Dementia
Espeland (2015)	USA (LIFE study)	Randomized controlled trial	NR	2 years	1602 (32.7)	70-89<br>78.8 (5.2)	ABI <0.9	13.2%	Cognition†<br>Impairment/ Dementia
Gardner (2021)	USA	Case-control Cross-sectional	NR	N/A	PAD: 58 (76)<br>Ctrl: 30 (30)	PAD: 69 (8.0)<br>Ctrl: 62 (8.0)	Ambulatory leg pain and ABI ≤0.9, and/or LER	66%	Cognition†
Guerchet (2013)	CAR, DRC (EDAC)	Prospective cohort*	2008-09	N/A	819 (38.7)	≥65<br>73.4 (6.5)	ABI ≤0.9	28.1%	Dementia
Guo (2023)	China	Case-control Cross-sectional	2019-21	N/A	CN: 259 (66.4)<br>SCD: 217 (61.8)	>50<br>CN: 63.2 (9.2)<br>SCD: 64.6 (10.3)	ABI ≤0.9	NR	Cognition†<br>Cognitive impairment
Gutierrez (2015)	USA (NHANES)	Prospective cohort*	1999-02	N/A	2573 (43.3)	≥60<br>70.5 (0.3)	ABI <0.9, difficulty or inability to walk ¼ mile	25.5%	Cognition†
Haan (1999)	USA (CHS)	Prospective cohort	1989-90	5-7 years	3333-4978 (NR)	≥65	ABI <0.9	NR	Cognition†
Hietanen	Finland	Prospective	1989	18 years	3859 (66.4)	50.0	ABI	NR	Dementia

(2013)		cohort			Dem: 123 (37)<br>No Dem: 3736 (67)	Dem: 63.7 (7.7)<br>No Dem: 49.9 (9.6)			
Hofman (1997)	Netherlands (Rotterdam Study)	Prospective cohort*	1990-93	N/A	1982 (35.6)	55-99	ABI <0.9	NR	Dementia
Johnson (2010)	UK (EAS)	Prospective cohort	1987-88	15 years	717 (NR)	55-74	ABI	NR	Cognition†
Kuller (2016)	USA (CHS/ CHS-CS)	Prospective cohort	1998-99	14 years	311 (35)	80.0	ABI <0.9	NR	Dementia
Laukka (2014)	UK (Lothian Birth Cohorts (1921/ 1936)	Prospective cohort*	1932 1947	N/A	PAD: 47 (45)<br>No PAD: 123 (50)<br>PAD: 91 (52)<br>No PAD: 657 (51)	PAD: 86.58 (.40)<br>No PAD: 86.62 (.40)<br>PAD: 72.47 (.68)<br>No PAD: 72.53 (.70)	ABI <0.9	28% (1931)<br>12% (1936)	Cognition†
Laurin (2007)	USA (HAAS)	Prospective cohort	1991-93	5 years	2588 (100)	71-93<br>76.9 (4.1)	ABI <0.9; 0.9 to 1.2; >1.20	10.5%	Dementia
Mangiafico (2006)	Italy	Case-control Cross-sectional	2003-04	N/A	APAD: 164 (74)<br>Ctrl: 164 (73)	APAD: 70.0 (3.4)<br>Ctrl: 70.3 (3.7)	ABI <0.9	50%	Cognition†
Moon (2015)	Korea (KLoSHA study)	Prospective cohort	2005-06	5 years	348 (50.9)	≥65<br>71.7 (6.3)	ABI	NR	Dementia
Muller (2007)	Netherlands	Cross-sectional	2001-02	N/A	396 (100)	40-80<br>Median: 60	ABI <0.9	NR	Cognition†
Newman (2005)	USA (CHS/ CHS-CS)	Prospective cohort	1992-94	5.4 years	2539 (39.9)	65-97<br>Median: 74	Self-report, PMH, test results, medication, ABI<0.9	PAD: 2.1%<br><0.9: 8.8%	Dementia
Pase (2016)	USA (FHS-OS)	Prospective cohort	1991-98	10 years	2063 (NR)<br>IDSBP <10: NR (46)<br>IDSBP ≥10: NR (43)	≥60<br>IDSBP<10: 71.5 (7.6)<br>IDSBP≥10: 72.5 (7.3)	ABI <0.9	NR	Dementia
Phillips (1997)	Canada	Case-control Cross-sectional	NR	N/A	PVD: 29 (72.4)<br>Ctrl: 30 (46.7)<br>CeVD: 29 (62.1)	PVD: 64.8 (11.3)<br>Ctrl: 68.3 (6.2)<br>CeVD: 66.1 (9.7)	ABI <0.8, LEA	________	Cognition†
Price (2006)	UK (EAS)	Prospective cohort	1987	10 years	717 (50.3)	55-74<br>63.6 (5.4)	ABI ≤0.95 and ≤0.90	16.5%	Cognition†
Reijmer (2011)	Netherlands (Hoorn Study)	Prospective cohort	2000-01	7 years	380<br>No MetS: 227 (52)<br>MetS: 153 (48)	60-87<br>No MetS: 67.8 (5.5)<br>MetS: 67.7 (5.4)	Rose questionnaire, ABI ≤0.9, arterial operation/ amputation	15.3%	Cognition†
Tarraf (2018)	USA (HCHS/SOL)	Prospective cohort*	2008-11	N/A	7991 (44.1)	45-74<br>55.9 (9.8)	ABI ≤0.9; 0.9 to <1; 1 to 1.39; ≥1.40	5.0%	Cognition†<br>Cognitive impairment
Tasci (2018)	Turkey	Case-control Cross-sectional	NR	N/A	352 (42.6)<br>Dem: 162 (43.2)<br>Ctrl: 190 (42.1)	78.91 (6.01)<br>Dem: 78.83 (6.14)<br>Ctrl: 78.97 (6.05)	ABI ≤0.9; 0.91 to 0.99; 1.0 to 1.4	Total: 25.0%<br>Dem: 35.2%<br>Ctrl: 16.3%	Dementia
VanOijen (2007)	Netherlands (Rotterdam Study)	Prospective cohort	1990-93	9 years	6,647 (40.7)	≥55<br>69.0 (8.8)	ABI <0.9	NR	Dementia
Wang (2016)	China (APAC Study)	Prospective cohort*	2012	N/A	3048 (56.7)	≥40<br>57.87 (11.08)	ABI <0.9	5.3%	Cognitive impairment

Weimar (2015)	Germany (HNR study)	Prospective cohort*	2006-08	N/A	1,732 (48.7)<br>MCI: 490 (46.9)<br>CN: 1,242 (49.4)	64.0 (7.6)<br>MCI: 66.1 (7.8)<br>CN: 63.1 (7.4)	ABI	NR	Cognitive impairment
Woo (2006)	China	Cross-sectional	NR	N/A	3998 (50.0)	≥65<br>72.5 (5.2)	ABI <0.9	6.9%	Cognitive impairment
Zimmerman n (2011)	USA (WALCS I and II)	Case-control Cross-sectional	2003-06	N/A	569<br>PAD: 335 (53.4)<br>Ctrl: 234 (41.9)	≥60<br>PAD: 74.46 (7.7)<br>Ctrl: 70.90 (7.3)	ABI <0.9	58.9%	Cognition†
Zuliani (2010)	Italy (InCHIANTI study)	Prospective cohort*	1998-2000	3 years	1051<br>Ctrl: 990 (44.2)<br>Dem: 61 (36.7)	65-102<br>Ctrl: 75 (7.2)<br>Dem: 85 (7.2	ABI <0.9	NR	Dementia

PAD= peripheral artery disease; N/A= not applicable; ABI= ankle brachial index; TSHA= Toledo Study of Healthy Ageing; ABCD= Nutrition, Cardiovascular Wellness and Diabetes study; ILSA= Italian Longitudinal Study on Aging; EDAC= Epidemiology of Dementia in Central Africa survey; CAR=Central African Republic; DRC= Republic of Congo; NHANES= National Health and Nutrition Examination Survey; CHS= Cardiovascular Health Study; CHS-CS Cardiovascular Health Cognition Study; EAS= Edinburgh Artery study; NR= not reported; HAAS= Honolulu-Asia Aging Study; FHS-OS= Framingham heart study original and offspring study; HCHS/SOL= Hispanic Community Health Study/Study of Latinos; HNR= Heinz Nixdorf Recall study; WALCS I and II= Walking and Leg Circulation study; PMH= Past medical history; Ctrl = controls; LER= lower extremity revascularization; Dem= dementia; APAD= asymptomatic peripheral artery disease; IDSBP= interarm differences in systolic blood pressure; PVD= peripheral vascular disease; LEA= lower-extremity amputation; CeVD= cerebrovascular disease; MetS= metabolic syndrome; APAC= Asymptomatic Polyvascular Abnormalities Community study; CI= cognitive impairment; MCI= mild cognitive impairment; CN= cognitively normal; SCD= Subtle cognitive decline; EPIDEMCA= Epidemiology of Dementia in Central Africa; LIFE= Lifestyle Interventions and Independence for Elders study; KLoSHA= Korean Longitudinal Study on Health and Aging study.

†= scores analysed on a continuous scale.

*= cross-sectional analysis only

Table S3. Risk of bias assessment using the Risk of Bias In Non-randomized Studies – of Exposures (ROBINS-E) tool

**Study**	**Sources of bias**							**Overall risk of bias**
	**Confounding**	**Classification and measurement of PAD**	**Selection**	**Post-exposure intervention**	**Missing data**	**Measurement of outcomes**	**Reporting**	
André-Petersson (2001)	🔴	🟢	🟢	🟢	🔴	🟢	🟡	🔴
Bareiro (2024)	🟢	🟡	🟡	🟢	🔴	🟢	🔴	🔴
Breteler (1994)	🔴	🟢	🟢	🟢	🟡	🟢	🟡	🔴
Bruunsgaard (1999)	🔴	🟢	🟡	🟢	🔴	🟢	🟡	🔴
Buscemi (2017)	🔴	🟢	🟡	🟢	🔴	🟢	🟡	🔴
Chen (2018)	🔴	🟢	🟢	🟢	🟡	🟢	🟡	🔴
Chen (2024)	🔴	🟢	🟢	🟢	🟢	🟢	🟡	🔴
Desormais (2018)	🔴	🟢	🟢	🟢	🔴	🟢	🟡	🔴
DiCarlo (2000)	🔴	🟢	🟢	🟢	🔴	🟢	🟡	🔴
DiCarlo (2007)	🔴	🟢	🟢	🟢	🔴	🟢	🟡	🔴
Espeland (2015)	🔴	🟢	🟢	🟢	🔴	🟢	🟡	🔴
Gardner (2021)	🟢	🟢	🟡	🟢	🟢	🟢	🟡	🟡
Guerchet (2013)	🟢	🟢	🟢	🟢	🟢	🟢	🟡	🟡
Guo 2023	🟡	🟢	🟡	🟢	🟢	🟢	🟡	🟡
Gutierrez (2015)	🟢	🟢	🟢	🟢	🔴	🟢	🟡	🔴
Haan (1999)	🟡	🟢	🟢	🟢	🟡	🟢	🟡	🟡
Hietanen (2013)	🔴	🟢	🟢	🟢	🟢	🟡	🟡	🔴
Hofman (1997)	🔴	🟢	🟢	🟢	🟡	🟢	🟡	🔴
Johnson (2010)	🔴	🟢	🟢	🟢	🔴	🟢	🟡	🔴
Kuller (2016)	🔴	🟢	🟢	🟢	🔴	🟢	🟡	🔴
Laukka (2014)	🟢	🟢	🟢	🟢	🔴	🟢	🟡	🔴
Laurin (2007)	🟢	🟢	🟢	🟢	🔴	🟢	🟡	🔴
Mangiafico (2006)	🔴	🟢	🔴	🟢	🟢	🟢	🟡	🔴
Moon (2015)	🔴	🟢	🟢	🟢	🔴	🟢	🟡	🔴
Muller (2007)	🔴	🟢	🟢	🟢	🟡	🟢	🟡	🔴

Newman (2005)	🟢	🟢	🟡	🟢	🔴	🟢	🟡	🔴
Pase (2016)	🔴	🟢	🟢	🟢	🔴	🟢	🟡	🔴
Phillips (1997)	🔴	🟢	🟢	🟢	🟢	🟢	🟡	🔴
Price (2006)	🔴	🟢	🟢	🟢	🔴	🟢	🟡	🔴
Reijmer (2011)	🔴	🟢	🟢	🟢	🔴	🟢	🟡	🔴
Tarraf (2018)	🟢	🟢	🟢	🟢	🔴	🟢	🟡	🔴
Tasci (2018)	🔴	🟢	🟡	🟢	🟢	🟡	🔴	🔴
VanOijen (2007)	🔴	🟢	🟢	🟢	🔴	🟢	🟡	🔴
Wang (2016)	🟢	🟢	🟢	🟢	🔴	🟢	🟡	🔴
Weimar (2015)	🟢	🟢	🟡	🟢	🔴	🟢	🟡	🔴
Woo (2006)	🔴	🟢	🟢	🟢	🟢	🟢	🔴	🔴
Zimmermann (2011)	🔴	🟢	🟢	🟢	🟡	🟢	🟡	🔴
Zuliani (2010)	🔴	🟢	🟢	🟢	🟡	🟢	🟡	🔴

🟢= Low risk of bias (little or no concern in this domain); 🟡= Some concerns (some concern in this domain, not clear that there is an important risk of bias);
🔴= High risk of bias (important problem in this domain).

Table S4. Cross-sectional and longitudinal associations between PAD and cognitive function domains

Study	Global	Language	Verbal Fluency	Visuospatial Ability	ST Memory	LT Memory	Processing Speed	S/M Function	Inhibition SA/SUSA	Cognitive Flexibility	Abstract Reasoning	Working Memory	Composite EF
**Cross-sectional associations between PAD and cognitive function domains**													
André-Petersson (2001)		Ⓤ		Ⓤ	Ⓤ		Ⓤ						
Bareiro (2024)	Ⓕ		Ⓕ	Ⓕ	Ⓕ	Ⓕ		Ⓕ					Ⓕ
Espeland (2015)	Ⓕ[c]				Ⓕ[c]	Ⓕ[c]	Ⓕ[c]		Ⓕ[c]	Ⓕ[c]		Ⓕ[c]	
Gardner (2021)					ⓊⓅⒻ	ⓊⓅⒻ	ⓊⓅⒻ			ⓊⓅⒻ		ⓊⓅⒻ	
Gutierrez (2015)							ⓊⒻ						
Laukka (2014)	ⓅⒻ		ⓅⒻ		ⓅⒻ	ⓅⒻ	ⓅⒻ		ⓅⒻ[b]		ⓅⒻ	ⓅⒻ	
Mangiafico (2006)				Ⓤ[a]	Ⓤ[a]	Ⓤ[a]	Ⓤ[a]			Ⓤ[a]		Ⓤ[a]	
Muller (2007)			Ⓟ		Ⓟ	Ⓟ	Ⓟ			Ⓟ		Ⓟ	
Phillips (1997)		Ⓤ[a]	Ⓤ[a]	Ⓤ[a]	Ⓤ[a]	Ⓤ[a]	Ⓤ[a]	Ⓤ[a]		Ⓤ[a]	Ⓤ[a]	Ⓤ[a]	
Tarraf (2018)	ⓊⓅⒻ		ⓊⓅⒻ		ⓊⓅⒻ	ⓊⓅⒻ	ⓊⓅⒻ						
**Longitudinal associations between PAD and cognitive function domains**													
Bareiro (2024)	Ⓕ		Ⓕ	Ⓕ	Ⓕ	Ⓕ		Ⓕ					Ⓕ
Espeland (2015)	Ⓕ[c]				Ⓕ[c]	Ⓕ[c]	Ⓕ[c]		Ⓕ[c]	Ⓕ[c]		Ⓕ[c]	
Haan (1999)	Ⓕ						Ⓕ						

Johnson (2010)<br>Performance 10yrs<br>Change (10-15yrs)	Ⓟ(green) Ⓟ(green)<br>Ⓟ(red) Ⓟ(red)												
Price (2006)			Ⓤ(green) Ⓟ(green) Ⓟ(red)		Ⓤ(red) Ⓟ(red) Ⓟ(red)	Ⓤ(red) Ⓟ(red) Ⓟ(red)	Ⓤ(green) Ⓟ(green) Ⓟ(green)				Ⓤ(green) Ⓟ(green) Ⓟ(red)		
Reijmer (2011)					Ⓟ(red)[d]	Ⓟ(red)[d]	Ⓟ(red)[d]						Ⓟ(red)

Ⓤ(red)=No association unadjusted analyses; Ⓟ(red)=No association partially adjusted analyses; Ⓕ(red)=No association fully-adjusted analyses; Ⓤ(green)=Association unadjusted analyses; Ⓟ(green)=Association partially adjusted analyses; Ⓕ(green)=Association fully-adjusted analyses; ST= short-term; LT= long-term; S/M= sensory/motor; SA= selective attention; SUSA= sustained attention; EF= executive function
[a=]age-education matched groups; [b=]aspects of inhibition, selection or sustained attention assessed as part of processing speed tasks; [c=]controlled for all factors except stroke; [d=]composite scores; [e=]men only.
